# Rational design of pyrazole–oxime hybrids as dual-targeting agents for glioma and breast cancer brain metastasis

**DOI:** 10.1007/s00210-026-05618-w

**Published:** 2026-06-27

**Authors:** Merve Gul, Mehmet Abdullah Alagoz, Ceylan Hepokur, Oztekin Algul

**Affiliations:** 1 Department of Pharmaceutical Chemistry, Faculty of Pharmacy, Inonu University, Malatya, Türkiye; 2 Department of Biochemistry, Faculty of Pharmacy, Sivas Cumhuriyet University, Sivas, Türkiye; 3 Department of Pharmaceutical Chemistry, Faculty of Pharmacy, Mersin University, Mersin, Türkiye; 4 Department of Pharmaceutical Chemistry, Faculty of Pharmacy, Erzincan Binali Yıldırım University, Erzincan, Türkiye

**Keywords:** Pyrazole derivatives, Breast cancer, CDK6, Apoptosis, SAR

## Abstract

To design, synthesize, and biologically evaluate a novel series of 1-(4-chlorophenyl)-2-(4-methylpyrazol-1-yl)ethenone oxime derivatives (**5–15**) as targeted anticancer agents, focusing on their selectivity for glioma, mechanistic action, and blood–brain barrier (BBB) permeability. The oxime derivatives were synthesized and characterized using ^1^H-NMR, ^13^C-NMR, and mass spectrometry. In vitro cytotoxicity was assessed against C6 (glioma), MCF-7 (breast cancer), and SH-SY5Y (human neuroblastoma) cell lines. Flow cytometry evaluated apoptosis and cell cycle arrest for the lead compound. Molecular docking simulations were performed against CDK6 (PDB: 5L2I) and P-glycoprotein/ABCB1 (PDB: 7A6E). All derivatives exhibited selective potency against C6 glioma cells. Compound **14** demonstrated the highest antiproliferative activity (IC₅₀: 31.23 µM) against tumor lines while sparing SH-SY5Y cells. Flow cytometry confirmed that compound **14** triggers apoptosis and induces cell cycle arrest in C6 cells. Molecular docking predicted the most favorable docking scores for compound **14** against both CDK6 and P-glycoprotein. Structure–activity relationships (SAR) analysis validates the 4-methylpyrazole core as a viable scaffold for targeted glioma therapy. These results suggest that further functionalization of the O-alkyl oxime moiety merits further investigation as a starting point for potentially brain-penetrant scaffolds, although *in vivo* and target-level validation are required.

**Supplementary Information:**

The online version contains supplementary material available at https://doi.org/10.1007/s00210-026-05618-w.

## Introduction

Gliomas represent the most common and aggressive primary tumors of the central nervous system (CNS), accounting for the majority of malignant brain tumors in adults (Louis et al. 2016). They are characterized by rapid cellular proliferation, diffuse infiltration into surrounding brain tissue, and high recurrence rates despite aggressive treatment strategies including surgical resection, radiotherapy, and chemotherapy (Benda et al. 1968). In parallel, secondary brain tumors arising from metastasis of extracranial cancers further complicate clinical management and significantly worsen patient outcomes. Among metastatic malignancies, breast cancer is one of the leading sources of brain metastases, with a substantial proportion of patients at advanced stages developing secondary CNS lesions (Lin et al. 2004; Niwńska et al. 2010; Lockman et al. 2010).

Breast cancer is the most frequently diagnosed malignancy among women worldwide and remains a major cause of cancer-related mortality (Bray et al. 2018). In its advanced stages, the disease commonly metastasizes to distant organs, including the brain, where therapeutic intervention is particularly challenging (Eichler et al. 2011). Notably, HER2-positive and triple-negative breast cancer subtypes exhibit disproportionately high incidences of CNS metastasis (Kennecke et al. 2010; Lin et al. 2012). Once brain involvement occurs, prognosis deteriorates dramatically due to limited treatment options and poor drug accessibility across the blood–brain barrier (BBB).

Although gliomas and breast cancer-derived brain metastases originate from distinct cellular lineages, their coexistence within the CNS highlights a critical therapeutic challenge. Importantly, these tumor types share several key pathological features, including aggressive proliferation, resistance to apoptosis, dysregulated survival signaling pathways, intrinsic or acquired drug resistance, and the necessity for therapeutic agents capable of penetrating the BBB (Lockman et al. 2010; Terceiro et al. 2023). These shared biological vulnerabilities suggest that a single molecular entity could be rationally designed to exert cytotoxic effects against both tumor types simultaneously. Based on these considerations, we hypothesized that small molecules incorporating multifunctional pharmacophores could act as dual-target anticancer agents, effectively inhibiting both primary glioma cells and metastatic breast cancer cells within the CNS. Rather than developing tumor-type-specific agents, this strategy aims to exploit common molecular and cellular dependencies, thereby offering a unified therapeutic approach for both primary and secondary brain tumors.

The development of effective therapeutic agents against both gliomas and breast cancer-derived brain metastases requires molecular architectures capable of addressing several shared pathological challenges, including uncontrolled proliferation, evasion of apoptosis, drug resistance, and limited drug accessibility across the BBB. Consequently, the design of small molecules intended for dual-target anticancer activity must integrate pharmacophores that are not only biologically potent but also structurally adaptable to meet these constraints.

**Fig. 1:**
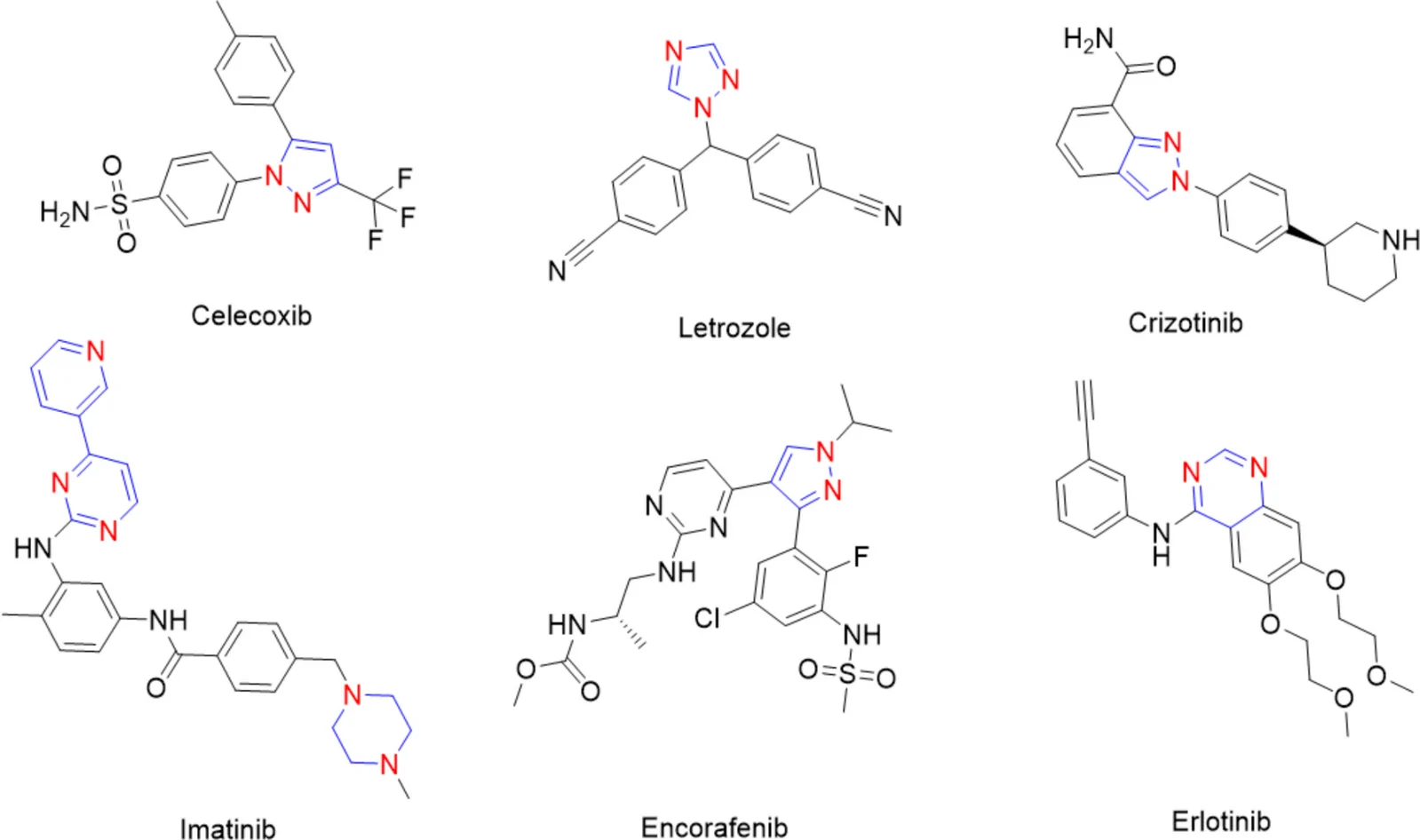
FDA-approved antineoplastic agents containing an azole scaffold

Pyrazole-based scaffolds were selected as a core structural element due to their well-documented ability to interact with molecular targets involved in cancer cell proliferation and survival. Pyrazole derivatives are frequently associated with inhibition of kinases, modulation of apoptotic pathways, and interference with oncogenic signaling cascades—mechanisms critically implicated in both glioma progression and breast cancer metastasis. The aromatic and planar nature of the pyrazole ring further facilitates favorable π–π stacking and hydrogen-bonding interactions with biological targets. Importantly, the incorporation of electron-donating or electron-withdrawing substituents on the pyrazole ring allows fine-tuning of electronic properties and biological activity, as evidenced by several clinically approved anticancer agents containing pyrazole or related azole motifs, including celecoxib, letrozole, and crizotinib (Fig. 1) (Ardevines et al. 2022; Kumar et al. 2023; Nehra et al. 2025; El-Gamal et al. 2022; Nitulescu et al. 2023; Bennani et al. 2020; Frampton 2013; Bhatnagar 2007).

Oxime functionalities were incorporated as complementary pharmacophores to enhance molecular flexibility and biological versatility. Oximes are known to exhibit anticancer and pro-apoptotic activities and possess the ability to act as both hydrogen bond donors and acceptors, thereby strengthening interactions with diverse biological targets. Furthermore, conversion of carbonyl groups into oxime moieties can significantly modulate lipophilicity and polarity, improve cellular uptake, and potentially facilitate BBB penetration—an essential requirement for agents targeting CNS tumors. Previous studies have demonstrated that oxime derivatives and their corresponding ethers or esters can improve pharmacokinetic behavior and target selectivity while maintaining or enhancing anticancer efficacy (Huang et al. 2018, 2019, 2017; Cui et al. 2019).

The strategic hybridization of pyrazole and oxime pharmacophores within a single molecular framework was therefore pursued to exploit the complementary advantages of both functionalities. Recent evidence suggests that such hybrid molecules can exert synergistic anticancer effects, surpassing the activity of individual pharmacophoric units, while simultaneously addressing drug resistance and BBB permeability limitations (Table 1) (Abdelrahman et al. 2023; Fadaly et al. 2020). By integrating a pyrazole core associated with antiproliferative and pro-apoptotic activity and an oxime moiety capable of enhancing molecular interactions and physicochemical properties, the designed compounds were anticipated to function as dual-target agents against both glioma cells and breast cancer cells with metastatic potential to the brain.

**Table 1:**
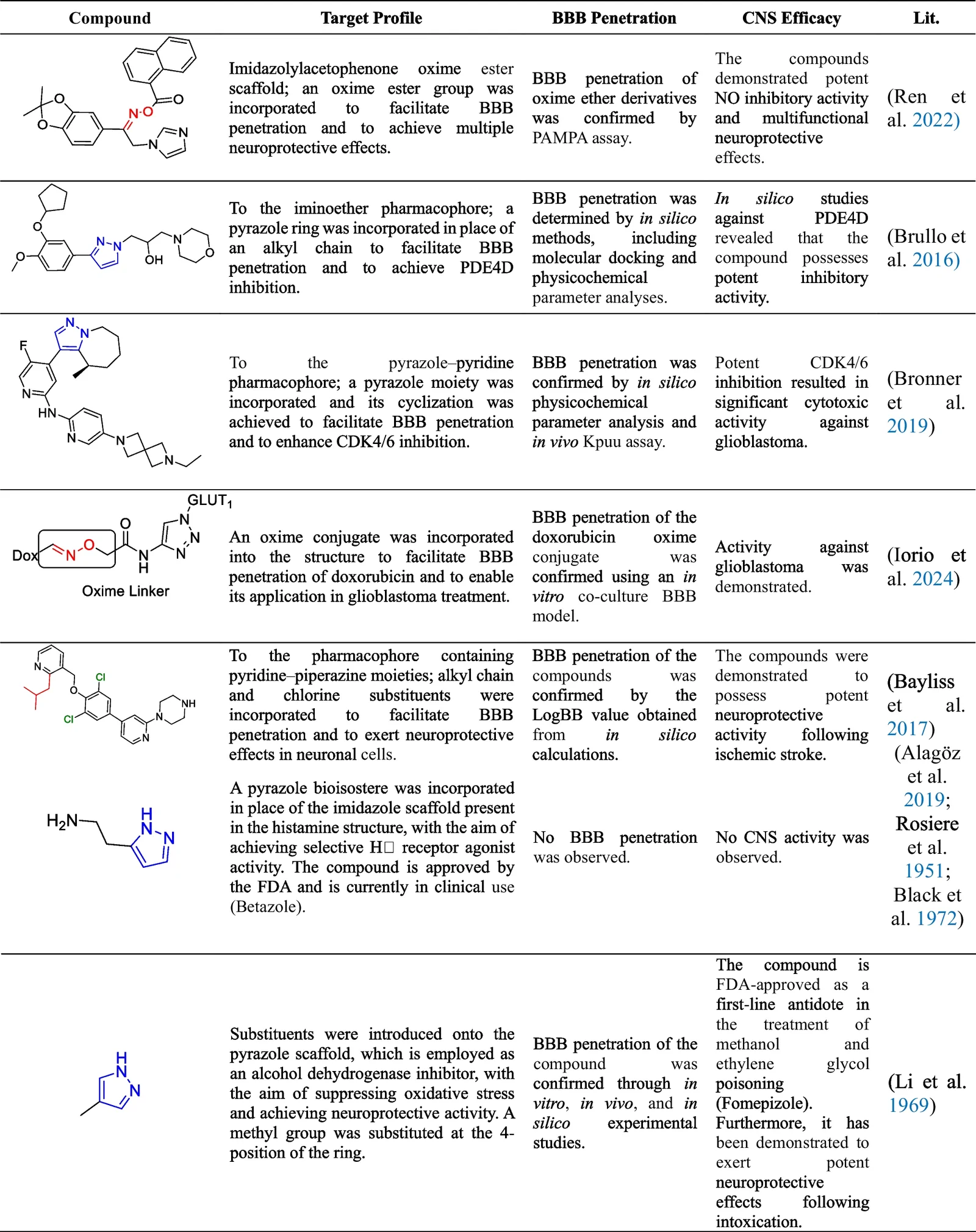
Critical effects of functional group modifications on the activity and stability of molecules reported in the literature

Literature reports have demonstrated the neuroprotective and cytotoxic potential of imidazolyl-acetophenone derivatives (Ren et al. 2022). Brullo and colleagues, while working on iminoether pharmacophores for PDE4 inhibition, incorporated a pyrazole ring to achieve BBB penetration, and reported that the addition of the pyrazole ring enabled compounds to cross the BBB (Brullo et al. 2016). In another study, the introduction of a pyrazole ring similarly facilitated BBB passage and enhanced CNS activity (Bronner et al. 2019). Iorio et al., in a study aimed at developing effective therapy for glioblastoma, synthesized an oxime-linked glycoconjugate of Doxorubicin to overcome the BBB barrier. *In vitro* results confirmed that the oxime linkage allowed Doxorubicin to cross the BBB (Iorio et al. 2024). Simple oxime derivatives containing a free hydroxyl group (− C = N–OH) can exhibit limited membrane permeability due to their polarity. To address this limitation, the free oxime was modified with oxime in ether functional groups, yielding a more rigid oxime structure. In CNS-targeted drug design, studies by Bayliss and colleagues confirmed the stability of the rigid oxime structure, preserving hydrogen bond donors while incorporating lipophilic alkyl chains, thereby increasing BBB permeability (Bayliss et al. 2017).

**Fig. 2:**
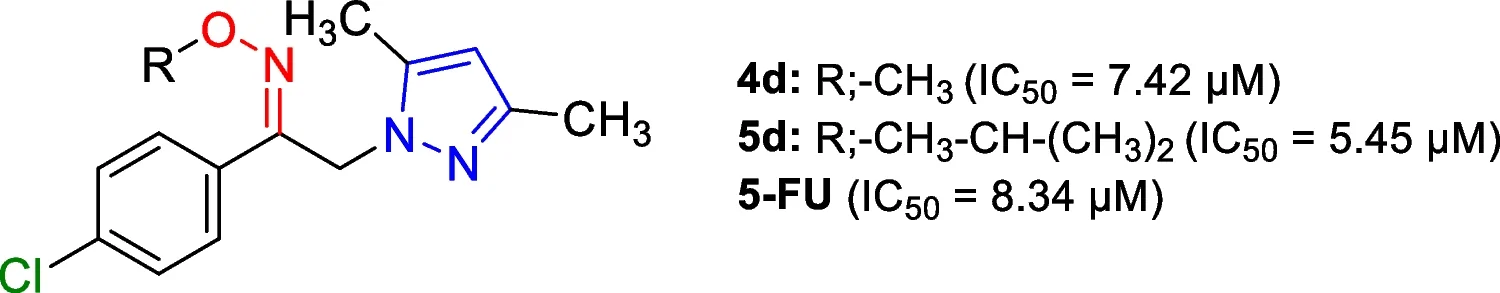
Derivatives containing a para-chloro ethanoneoxime moiety with cytotoxic activity against glioma

In our previous study, a series of 1-aryl-2-(3,5-dimethylpyrazol-1-yl)ethanoneoxime ether derivatives (Fig. 2) were synthesized and found to exert significant effects on glioma cells. Among these compounds, derivatives bearing a para-chloro group on the aryl ring exhibited lower IC₅₀ values than the reference compound 5-fluorouracil (5-FU) (Alagöz et al. 2019).

The promising results obtained from our previous study prompted further investigation into the effects of substitution patterns on the pyrazole scaffold with respect to BBB permeability and biological activity. In the literature, the monosubstituted pyrazole derivative betazole (an FDA-approved antihistamine) fails to cross the BBB due to its polar structure and consequently exhibits no CNS activity (Rosiere and Grossman 1951; Black et al. 1972). This observation underscores the critical role of logP balance in the design of our compounds. To overcome the BBB penetration issue and to expand the series beyond the 3,5-dimethylpyrazole core used in our previous work, the present study employs the 4-methylpyrazole (fomepizole) nucleus as the pharmacophoric group. Fomepizole is an FDA-approved antidote and is the firstline treatment for methanol and ethylene glycol poisoning in emergency settings (Li and Theorell 1969). The rationale for selecting fomepizole lies in the lipophilic contribution of the methyl group at the 4-position, which supports BBB penetration; the potential neuroprotective effects of the pyrazole core on the CNS; and its monosubstituted, small-volume structure that does not introduce steric hindrance (Akakpo et al. 2020; Yu et al. 2021). Although fomepizole is established as an antidote, its derivatization with oxime ether functional groups and evaluation of its activity against glioma represent a chemical space that has not yet been systematically explored in the literature. Accordingly, the present study also aims to fill this gap by providing data on structure–activity relationships for fomepizole derivatives, an area that remains largely unexplored.

Based on the information gathered from the literature, this study designed compounds expected to exert potent cytotoxic effects against both breast cancer and glioma. The compounds were designed as hybrid molecules combining a pyrazole ring with an acetophenone core. The hydroxyl group in the oxime structure was converted to oxime ethers using alkyl chains, thereby preserving chemical stability while optimizing the lipophilic character required for the anticancer agent to reach the target site. This rational design strategy directly aligns with the central hypothesis of the present study, aiming to generate multifunctional small molecules capable of simultaneously targeting shared vulnerabilities in primary and secondary brain tumors.

Guided by this hypothesis and design rationale, we synthesized a series of eleven novel 1-(4-chlorophenyl)−2-(4-methylpyrazol-1-yl)ethenone oxime derivatives (compounds 5–15). These compounds were conceived as hybrid structures integrating the complementary biological advantages of both pyrazole and oxime pharmacophores within a single molecular entity. To evaluate their dual-target potential, the antiproliferative activities of the synthesized compounds were systematically assessed against rat C6 glioma cells (representing primary CNS tumors) and human MCF-7 breast cancer cells (representing extracranial malignancies with high metastatic propensity to the brain). In parallel, cytocompatibility studies were conducted using SH-SY5Y human neuroblastoma cells as a neuronal reference line to assess selectivity toward cancer cells.

To elucidate the underlying mechanisms responsible for the observed biological activities, structure–activity relationship (SAR) analyses and flow cytometry-based apoptosis assays were performed. Molecular docking and molecular dynamics simulation studies were conducted to evaluate the mechanism of action of the compounds on the CNS and to elucidate the BBB transit mechanisms. Furthermore, molecular docking studies were employed to predict the binding interactions of the synthesized compounds with relevant oncogenic targets implicated in tumor proliferation and survival. To further validate these interactions at the atomistic level, molecular dynamics (MD) simulations were conducted to assess the conformational stability and binding persistence of the most potent candidates within the active sites over time. Collectively, this integrated experimental and computational approach provides suggests that the rationally designed pyrazole–oxime hybrid compounds described herein possess significant potential as dual-target anticancer agents against glioma and breast cancer, warranting further investigation.

## Results and discussion

### Chemistry

**Scheme 1:**
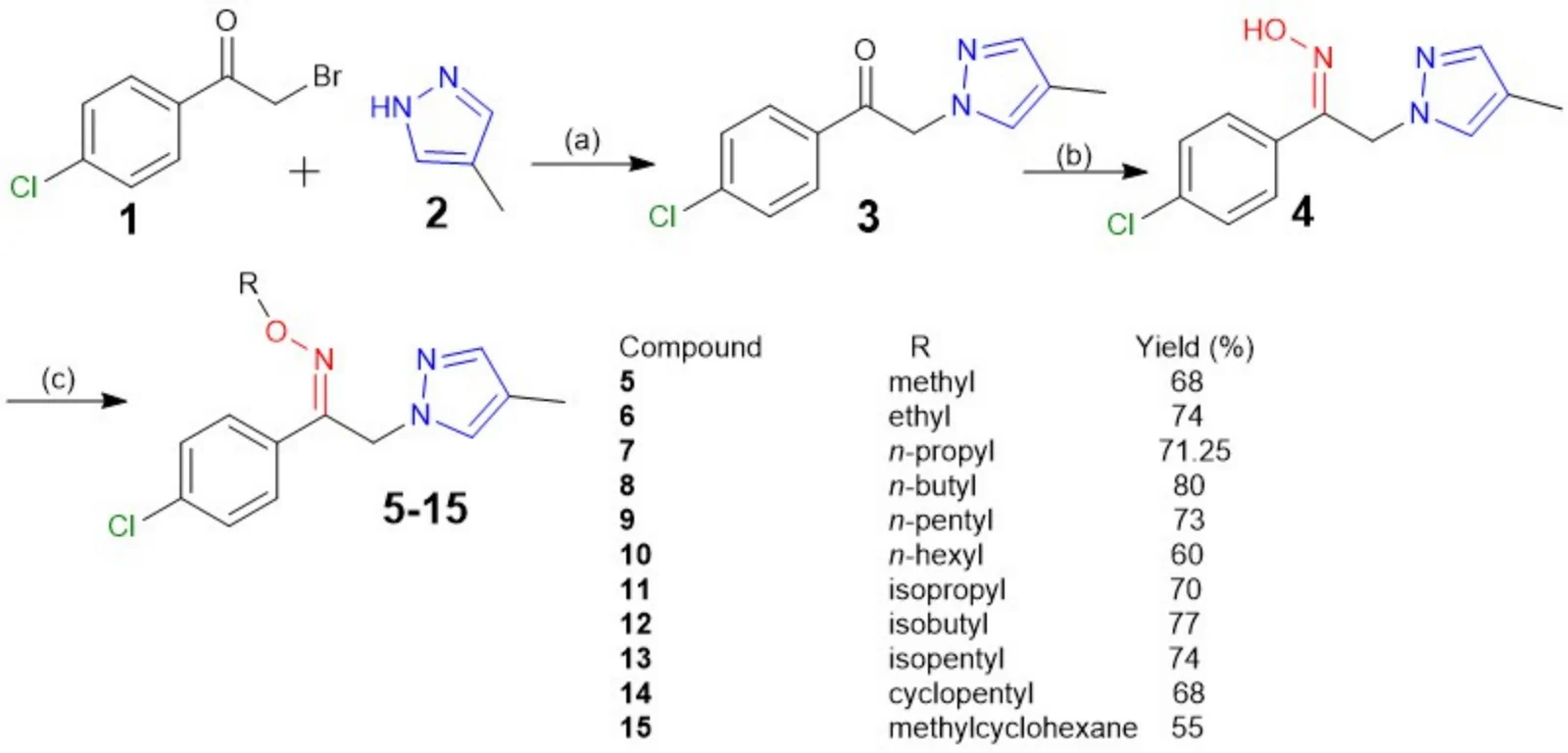
Synthesis of novel pyrazole-acetophenone oxime ether derivatives (**5–15**) Reagents and conditions: (**a**) DMF, 0 ℃- 2 h/rt-22 h; (**b**) NH_2_OH.HCl, 15 N NaOH/Ethanol, reflux-2 h; (**c**) NaOEt reflux-1 h

The starting material, 1-(4-chlorophenyl)−2-(4-methyl-1H-pyrazole-1-yl)ethanol (**3**), was synthesised by the N-alkylation method (a). The reason for choosing this method was the failure of our research group's previous attempts to obtain similar 1-aryl-2-(1*H*−3,5-dimethylpyrazole-1-yl)ethanol compounds by ring-closing reaction; in these attempts, the reaction could not be completed, and low yields were obtained. The N-alkylation method, however, overcame these limitations and gave the ketone product with an average yield of 74% (Scheme 1) (Alagöz et al. 2019).

The obtained ketone compound (**3**) was reacted with hydroxylamine hydrochloride in a basic environment and converted to an oxime derivative (**4**) in step (b). This step was carried out with a yield of 66%. Subsequently, the O-alkylation step (c) was performed; as a result of reactions carried out using suitable alkyl halides, 11 new oxime ether derivatives (**5–15**) were obtained. The products were purified by preparative thin-layer chromatography (ethyl acetate/n-hexane, 2:1).

When the yields of the o-alkylation reactions (c) are examined, it is seen that the highest yield is obtained with compound **8** using n-butylbromide, with 80%. The lowest yields were recorded as 60% for bromohexane (compound 10) and 55% for bromomethylcyclohexane (compound 15). A significant decrease in yield was observed when the alkyl chain length increased (C ≥ 5) and when cyclic groups were included in the structure; this is thought to be due to the steric hindrance created by bulky and cyclic groups, which makes the nucleophilic substitution reaction more difficult. The structural elucidation of the synthesized compounds was carried out using spectroscopic methods. In the ^1^H-NMR spectra, the protons forming the main backbone of the compounds gave signals at the expected chemical shift values. The protons in the pyrazole ring were observed as two symmetrical doublets in the range of 7.11–7.22 ppm, and those in the phenyl ring in the range of 7.20–7.60 ppm. The structural differences between the compounds stem from the alkyl chains attached to the oxygen atom of the oxime group, and the number and symmetrical structure of the protons belonging to these chains were confirmed via the aliphatic region (0.00–2.00 ppm). The protons of the methyl group (CH₃) at position 4 of the pyrazole ring were observed as singlets in the range of 1.94–1.97 ppm. In the ^13^C-NMR spectra, it was observed that the carbons of the 4-methylpyrazole and 4-chlorophenyl rings, which are common in all compounds, exhibited similar chemical shift values; it was determined that the aromatic carbons resonated in the range of 110–159 ppm. The carbon signal corresponding to the methyl group on the pyrazole ring was observed at approximately 7–8 ppm, while the methylene (–CH₂–) carbon bridging the phenyl and pyrazole rings was observed at 44–46 ppm. The structural diversity within the series was supported by the number of signals and chemical shift values ​​of the alkyl chains of the oxime ether derivatives. Carbon signals belonging to the C = N–O– group were recorded in the range of 54–80 ppm; the carbon directly bonded to the oxygen atom (C = N–O–CH–) gave a signal of 62–86 ppm, and the other aliphatic carbons in the alkyl chain gave signals in the range of 0–35 ppm. The [M + H]⁺ peaks of the compounds were included in the mass spectra; it was observed that the calculated and measured values ​​coincided with each other. The observation of two peaks of different intensities corresponding to the same ion, due to the natural isotopic distribution of the chlorine atom in the structure (~ 3:1 ratio), was also confirmed through mass spectrometry (Meija et al. 2016). In the HRMS analysis of compound 14, molecular ion peaks were obtained as 322.1494 (25%) and 320.1524 (75%) (Figure S30).

### Biological activity studies

#### Cytotoxic activity assay

To evaluate the dual-target anticancer potential of the synthesized pyrazole–oxime hybrids, their cytotoxic activities were assessed against two biologically relevant cell lines: MCF-7 (human breast adenocarcinoma), representing an extracranial malignancy with high metastatic propensity to the brain, and C6 (rat glioma), representing a primary CNS tumor. Cytocompatibility was assessed using SH-SY5Y human neuroblastoma cells as a neuronal reference line to calculate selectivity indices (SI = IC₅₀ on SH-SY5Y/IC₅₀ on cancer cell line), where higher SI values indicate greater selectivity greater selectivity relative to the SH-SY5Y neuronal reference line (a neuroblastoma-derived line, not a primary normal neuron).

The use of C6 and MCF-7 cell lines in the cytotoxicity assay was based on the aim of evaluating the anticancer potential of the synthesized compounds in two biologically distinct tumor models. C6 glioma cells were selected as a primary brain tumor-related glial cell model, while MCF-7 breast cancer cells were included as a representative epithelial cancer model with relevance to metastatic disease, since breast cancer is one of the major malignancies associated with brain metastasis. Thus, the combined use of these two cell lines enabled the assessment of compound activity against both primary brain tumor-like cells and a cancer cell type clinically associated with secondary brain involvement. The inclusion of SH-SY5Y neuronal cells further allowed the estimation of tumor selectivity and potential neurotoxicity.

The cytotoxicity results (IC₅₀ values in µM) and selectivity indices for all synthesized compounds (**5–15**) and the reference drug Doxorubicin are summarized in Table 2.

**Table 2:** Cytotoxic effects (IC₅₀, µM) and selectivity indices (SI) of compounds **5–15** and Doxorubicin on MCF-7, C6, and SH-SY5Y cell lines

Compound	IC_50_ (µM)			SI*	
	MCF-7	C6	SH-SY5Y	C6	MCF-7
**5**	59.53 ± 3.72	41.18 ± 1.29	166.04 ± 3.30	4.03	2.79
**6**	33.30 ± 0.83	29.74 ± 2.41	186.39 ± 0.83	6.27	5.60
**7**	26.49 ± 1.54	22.41 ± 0.82	111.52 ± 2.67	4.98	4.21
**8**	74.92 ± 2.45	61.90 ± 0.75	107.55 ± 1.83	1.74	1.44
**9**	65.10 ± 2.66	59.37 ± 2.09	125.82 ± 2.38	2.12	1.93
**10**	77.04 ± 1.68	54.99 ± 0.69	124.03 ± 2.55	2.26	1.61
**11**	109.23 ± 0.79	51.10 ± 2.60	179.96 ± 2.91	3.52	1.65
**12**	153.27 ± 1.05	163.14 ± 2.91	171.06 ± 2.81	1.05	1.12
**13**	149.17 ± 2.38	139.95 ± 2.97	166.49 ± 2.28	1.19	1.12
**14**	43.17 ± 2.45	31.23 ± 0.35	283.59 ± 3.02	9.02	6.57
**15**	45.48 ± 0.98	25.10 ± 1.24	102.67 ± 2.46	4.09	2.26
Doxorubicin	5.08 ± 0.06	58.43 ± 1.80	139.59 ± 1.99	2.39	27.48

*SI values are calculated as IC₅₀ (SH-SY5Y)/IC₅₀ (cancer cell line). Higher SI indicates greater selectivity. Data are presented as mean ± SD. Statistical significance was evaluated compared with doxorubicin within the same cell line. *p < 0.05, **p < 0.01

Overall, the synthesized compounds exhibited higher cytotoxicity toward C6 glioma cells compared to MCF-7 breast cancer cells, as reflected by the generally lower IC₅₀ values and consequently higher SI values for the C6 cell line. For instance, compounds **6**, **7**, and **11** showed SI values of 6.27, 4.98, and 3.52 for C6, respectively, while their corresponding SI values for MCF-7 were lower (5.60, 4.21, and 1.65). These results suggest that the pyrazole–oxime hybrids possess a stronger selective antiproliferative effect against glioma cells.

**Fig. 3:**
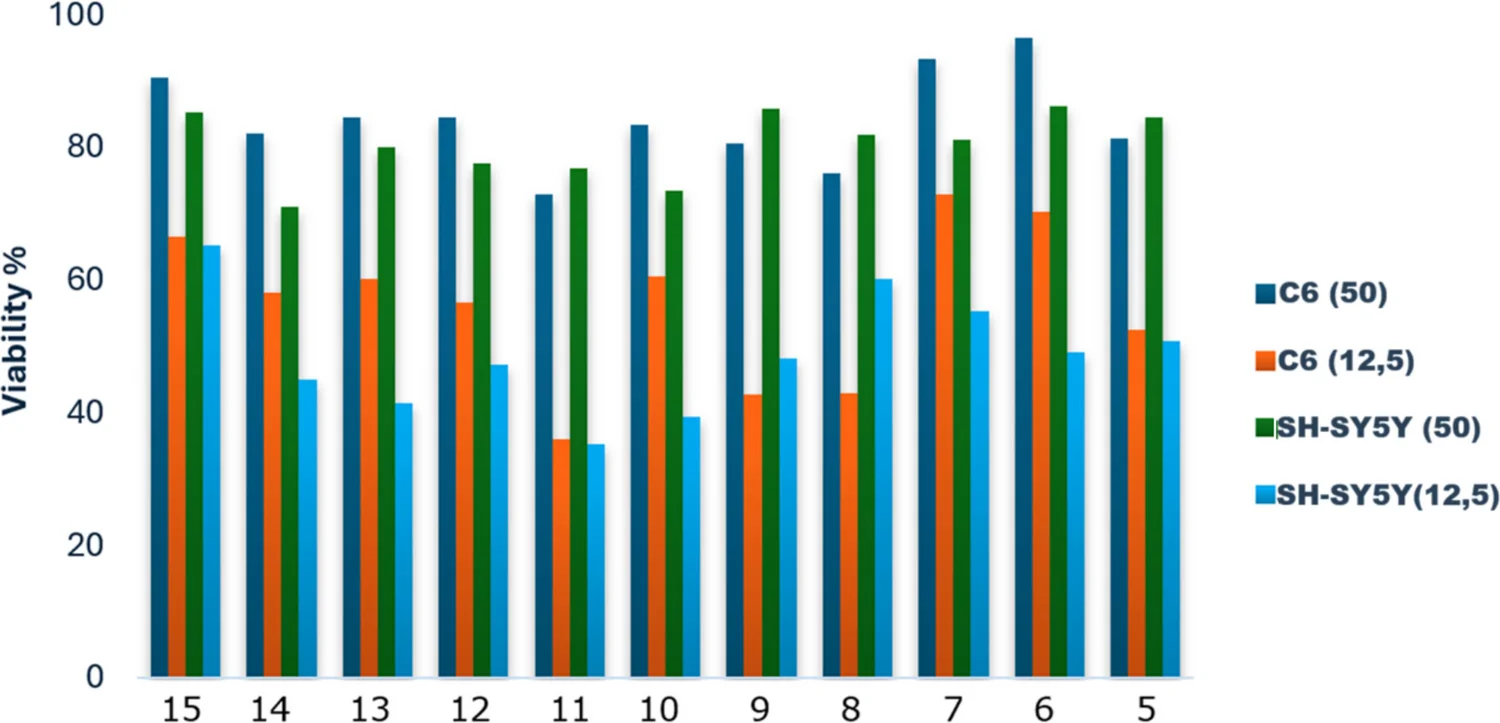
Illustrates the cell viability (%) of MCF-7 and C6 cell lines following treatment with selected compounds at concentrations of 50 and 12.5 µg/mL

Although compound **7** exhibited a slightly lower IC₅₀ value against C6 cells (22.41 µM) compared to compound **14** (31.23 µM), the SI represents a more clinically relevant parameter for candidate prioritization, as it reflects the therapeutic window between anticancer potency and potential neurotoxicity. Compound **14** demonstrated the highest SI value for C6 cells (9.02) in the entire series, indicating a substantially wider safety margin. Consistent with the IC₅₀ and SI data, compound **14** exhibited the highest selectivity and the strongest antiproliferative effect in the C6 cell line, further supporting its potential as a lead candidate for dual-target therapy against primary and metastatic brain tumors **(**Fig. 3**)**.

#### Flow cytometry analysis

To elucidate the mechanism of cell death induced by the most promising compound, flow cytometry analysis was performed using annexin V/propidium iodide (PI) double staining. This method allows discrimination between viable, early apoptotic, late apoptotic, and necrotic cell populations. Compound **14**, which exhibited the highest selectivity index against C6 glioma cells, was selected for further mechanistic evaluation. Cells were treated with compound **14** at its respective IC₅₀ concentration for 24 h and then analyzed by flow cytometry.

**Fig. 4:**
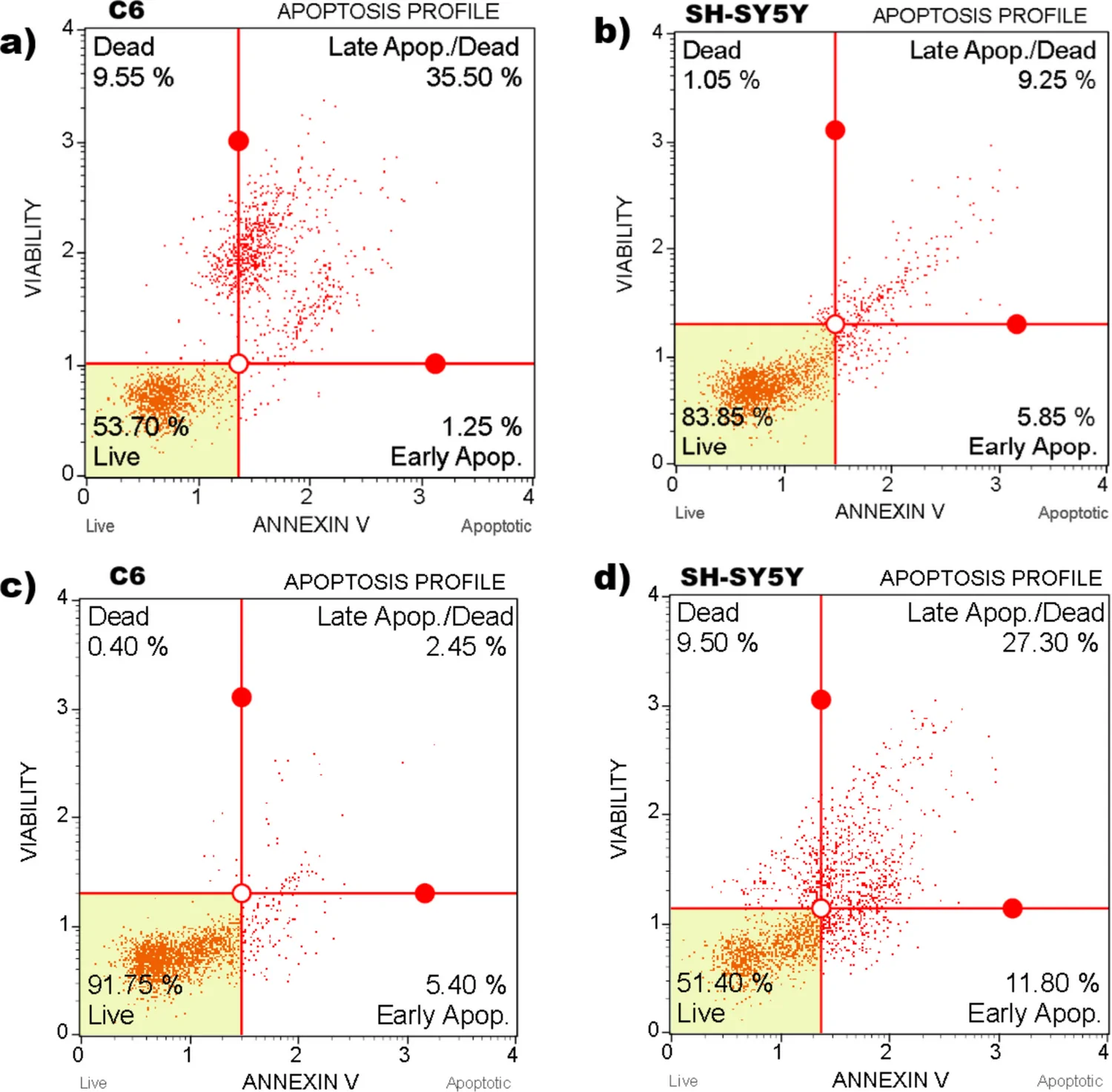
Flow cytometry analysis of apoptosis induced by compound **14** (**a**, **b**) and Doxorubicine (**c**, **d**). Apoptosis profiles of C6 glioma cells and SH-SY5Y neuronal cells following treatment with compound **14** and Doxorubicine at its IC₅₀ concentration

Figure 4 presents the apoptosis profiles of C6 and SH-SY5Y cells following treatment with compound **14** at its IC₅₀ concentration. The results demonstrate a marked increase in both early and late apoptotic populations in C6 cells, whereas SH-SY5Y cells (neuronal reference line) showed significantly lower levels of apoptosis under the same conditions. This differential response is consistent with the high SI (9.02) calculated for compound **14** on C6 cells and further supports its selective pro-apoptotic activity toward glioma cells.

**Fig. 5:**
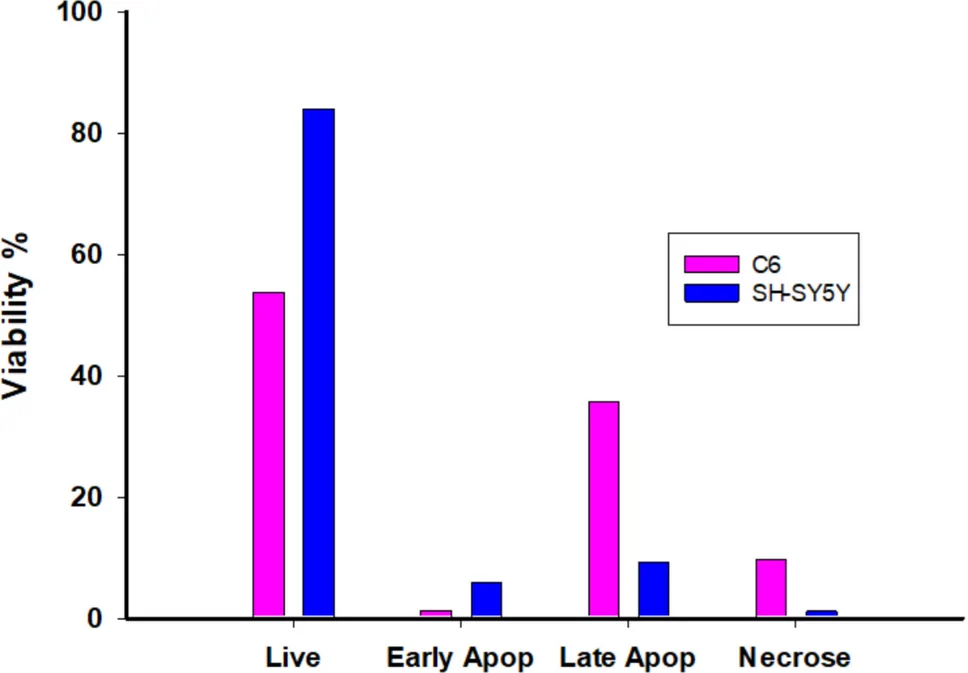
Quantitative distribution of live, early apoptotic, late apoptotic, and necrotic cell populations in C6 and SH-SY5Y cells following treatment with compound **14** at its IC₅₀ concentration

Figure 5 provides a quantitative summary of the flow cytometry data, showing the distribution of live, early apoptotic, late apoptotic, and necrotic cell populations in both cell lines. The data clearly indicates that compound **14** predominantly induces apoptosis rather than necrosis in C6 cells, with a substantial proportion of cells in early and late apoptotic stages. In contrast, SH-SY5Y cells remained largely viable, confirming the preferential cytotoxicity of compound **14** toward cancerous C6 cells over non-malignant neuronal cells.

**Fig. 6:**
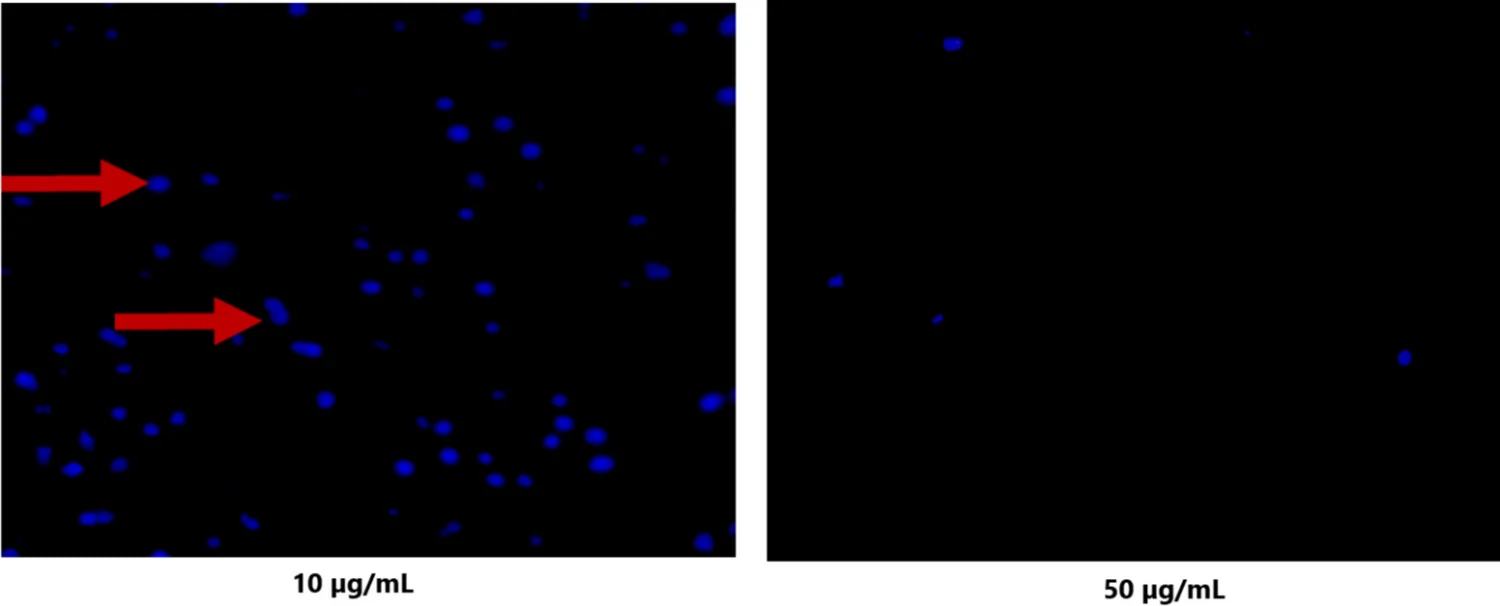
DAPI staining of C6 glioma cells treated with compound **14** at **10** and 50 µg/mL. At 10 µg/mL, nuclear alterations compatible with cell death were observed, whereas at 50 µg/mL, a marked reduction in DAPI-positive nuclei was evident. This finding may reflect extensive cell death and/or cell detachment; therefore, DAPI staining was interpreted as supportive morphological evidence rather than a stand-alone confirmation of apoptosis

To obtain direct morphological evidence of apoptosis, DAPI (4′,6-diamidino-2-phenylindole) nuclear staining was performed. DAPI binds specifically to AT-rich regions in the minor groove of double-stranded DNA, emitting bright blue fluorescence upon binding. Under normal physiological conditions, DAPI cannot penetrate the intact plasma membrane of viable cells; therefore, it selectively stains dead, necrotic, or fixed cells, allowing clear visualization of nuclear morphology. As shown in Fig. 6, DAPI staining of C6 cells treated with compound **14** revealed characteristic apoptotic nuclear changes, including chromatin condensation, nuclear fragmentation, and formation of apoptotic bodies (indicated by red arrows). Untreated control cells exhibited uniformly stained, intact nuclei with normal morphology. These physical changes provide unequivocal evidence of apoptosis.

Collectively, the flow cytometry results, together with the supportive DAPI staining findings, suggest that compound **14** induces cell death in C6 glioma cells, at least partly through apoptotic pathways. However, the pronounced reduction in DAPI-positive nuclei observed at higher concentrations should be interpreted cautiously, as it may reflect extensive cell death and/or cell detachment rather than apoptosis-specific nuclear morphology alone. In contrast, compound **14** showed relatively limited cytotoxicity toward normal SH-SY5Y neuronal cells, supporting its potential selectivity and warranting further investigation as a pyrazole–oxime hybrid with anticancer activity against brain tumor cells.

#### Cell cycle analysis

Cancer cells are characterized by rapid and uncontrolled proliferation, which drives the progression of malignant tumors. Dysregulation of cell cycle progression is a fundamental hallmark of tumor development. The cell cycle is tightly regulated by two major checkpoints: the G1/S and G2/M phases.

Most cell cycle disruptions occur during the G1 phase, a critical stage where multiple signals influencing cell division and cellular development programs are integrated. When DNA damage cannot be adequately repaired, the G2 checkpoint prevents cells from entering mitosis, thereby providing an opportunity for repair mechanisms to restore genomic integrity (Sumiya 2021).

To investigate whether the cytotoxic effect of compound **14** is mediated through cell cycle interference, its effect on the cell cycle distribution of C6 cells was analyzed by flow cytometry following PI staining.

**Fig. 7:**
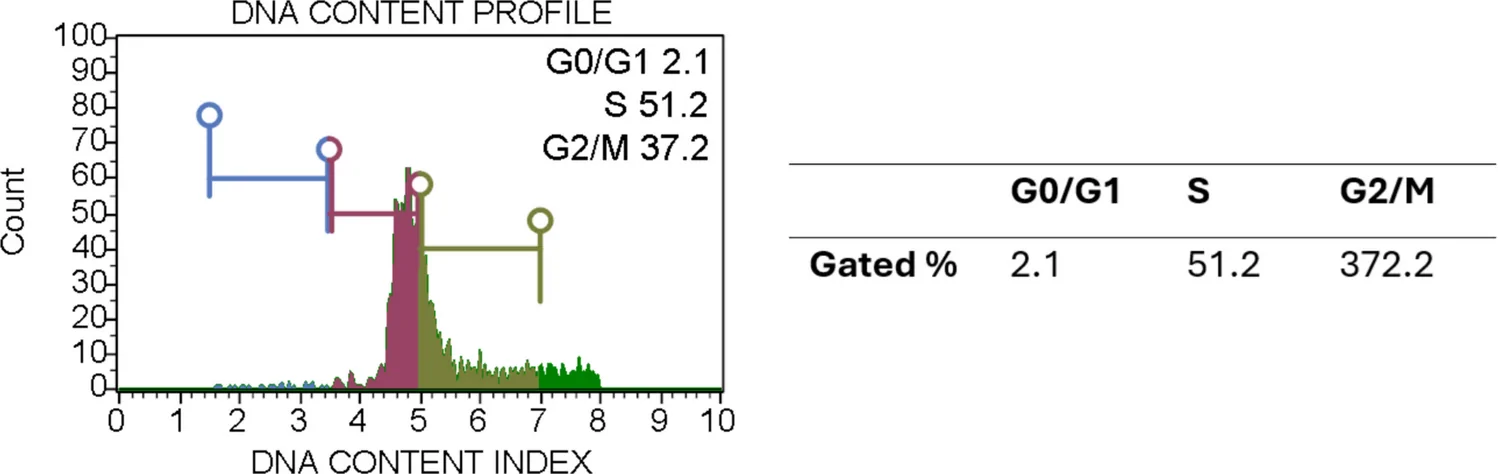
Cell cycle distribution of C6 cells following treatment with compound **14**. Treatment at the IC₅₀ concentration resulted in G2/M-phase arrest

Figure 7 shows the DNA content profile of C6 cells treated with compound **14** compared with the untreated/vehicle-treated control group. Treatment with compound **14** was associated with an increased accumulation of cells in the G2/M phase, together with a reduction in the G0/G1 and S phase populations. These findings suggest that compound **14** may interfere with cell cycle progression and induce G2/M phase arrest in C6 glioma cells. However, further experiments using multiple concentrations are required to confirm the dose-dependent effect of compound **14** on cell cycle regulation.

Consistent with the cell cycle arrest, the MTT assay demonstrated that compound **14** significantly reduced C6 cell viability in a dose-dependent manner, with an IC₅₀ value of 31.23 ± 0.35 µM. Furthermore, flow cytometry analysis of apoptosis (as described in Sect. "Flow cytometry analysis") revealed that compound **14** increased both early and late apoptotic populations, consistent with cytotoxicity mediated through the induction of apoptosis. DAPI nuclear staining provided visual confirmation of these findings, showing characteristic apoptotic morphological changes, including nuclear fragmentation and chromatin condensation, in compound **14**-treated C6 cells.

Taken together, these results demonstrate that compound **14** exerts its antiproliferative effect against C6 glioma cells by inducing G2/M phase arrest and subsequently triggering apoptotic cell death.

### *In silico* studies

GBM is among the most malignant types of cancer, and there is currently no definitive treatment. Literature shows that glioma-associated microglia (GAM) plays a decisive role in tumour formation and progression in GBM (Lin et al. 2023; Hambardzumyan et al. 2016). Molecular docking and MD simulation studies were carried out to investigate the mechanisms of action of the synthesised oxime derivatives in more detail. The modelling approach was based on two main stages:

- I - Evaluation of the possible inhibitory effects of the compounds on the GBM-associated CDK6 protein (PDB ID: 5L2I) and
- II – Investigation of the interactions of the compounds with the P-glycoprotein (P-gp/ABCB1; PDB: 7A6E), which plays a critical role in BBB transit.

#### Molecular docking

CDK6 antigen levels have been shown to be higher in tumour tissue compared to healthy brain tissue, and this increase has been associated with poor survival. It has been shown that CDK6 levels are elevated in temozolomide-resistant (TMZ-R) GBM samples; CDK6 inhibition via Palbociclib suppresses tumour growth in these resistant models and is linked to the oncogenic activity of CDK6 (Michaud et al. 2010). CDK6 is a novel target for GBM-focused drug development studies; CDK6 expression and activity are increased in GBM cells, and CDK6 inhibitors can significantly suppress GBM both *in vitro* and *in vivo* (Michaud et al. 2010; Li et al. 2017; Olmez et al. 2017). In TMZ-R cells treated with the CDK4/6 inhibitor Palbociclib, a significant decrease in M2-GAM production capacity was observed (Li et al. 2019). FDA-approved CDK4/6 inhibitors include Palbociclib, Abemaciclib, and Ribociclib; In addition to their established uses in the treatment of HR +/HER2 − breast cancer, Palbociclib is also being considered in clinical trials as a potential chemotherapeutic agent in primary and secondary brain tumours (Fassl et al. 2022). Clinical studies have confirmed that Palbociclib can cross the BBB, and it has been emphasised that this crossing may be a decisive criterion in the treatment efficacy against glioma tumours. For this reason, considering the importance of BBB crossing in glioma tumours, a second modelling study was conducted through the P-gp target to investigate the crossing potential of compounds. The P-glycoprotein (P-gp; ABCB1/MDR1), a multidrug transporter, plays a critical role in cellular detoxification by expelling foreign compounds through the efflux system. This mechanism can directly affect the pharmacokinetics of many drugs and can pave the way for the development of chemotherapy resistance in cancer cells (Koltai 2022). P-gp is more than just a passive barrier. In the context of tumour formation and protection against neurotoxic agents, it functions as a detoxification system that dynamically restricts brain exposure to substrates (Tournier et al. 2019, 2021). P-gp, which is highly expressed on various tissue surfaces, especially brain capillary endothelial cells, intestinal epithelium, liver, and the blood–brain barrier, stands out as one of the key efflux components of the BBB (Paul et al. 2026). P-gp recognises lipophilic molecules located in the membrane, recruits them to transmembrane binding pockets, and pumps these molecules back out of the cell, to the blood side, thanks to ATP-dependent conformational changes (Yamasaki et al. 2022). PET studies in humans and animals have shown that the brain distribution of certain substrates increases when ABCB1 inhibitors are applied. For example, in PET studies performed with metoclopramide, it was reported that ABCB1 inhibition increased VT and K1 values. Suppression of P-gp function, whether due to genetic variants or inhibitor use, can significantly increase brain exposure for some substrates. Strong P-gp inhibitors such as Tariquidar and Elacridar have been shown to increase the brain accumulation of certain substrates. In oncological agents such as Temozolomide, it has been reported that ABCB1/ABCG2 inhibition can enhance brain penetration and antitumor efficacy; however, it can also increase the risk of pharmacokinetic and toxic interactions of inhibitors (Liubota et al. 2018; Bauer et al. 2019; Lentzas et al. 2024).

In this study, it is assumed that oxime derivatives act primarily as P-gp modulators or inhibitors, not as P-gp substrates. Docking studies against the 7A6E protein were performed to evaluate whether the compounds have P-gp inhibitor properties.

When the results obtained from the docking studies were examined (Table 3), it was observed that compound **14** exhibited the closest score to Palbociclib with a value of −6.759 kcal/mol when compared with the docking score of cocrystal Palbociclib in the CDK6 (5L2I) structure (−9.011 kcal/mol). Compounds **12** and **13** were determined to be the compounds with the lowest docking scores, with −5.520 and −5.555 kcal/mol, respectively. In ligand–protein interaction in the 5L2I structure, two basic types of interaction, hydrophobic and hydrophilic, play a decisive role (Chen et al. 2016). These residues are associated with CDK6 inhibition in the literature shape selectivity and function in the regulation of ligand–protein interactions (Shi et al. 2022). According to the literature findings, it has been reported that electrostatic interactions occur with Glu61, Gln103 and Asp163 residues in the polar region, while hydrophobic interactions occur with Ile19, Val27, Ala41, Phe98 and Leu152 residues (Shi et al. 2022; Roskoski 2019). In addition, Val101 is identified as an important residue that acts as a structural backbone in inhibition (Chen et al. 2016; Shi et al. 2022; Roskoski 2019).

**Table 3:** Docking scores of designed compounds and reference compounds to 7A6E and 5L2I (kcal/mol)

Compounds	7A6E (ABCB1)	5L2I (CDK6)
**5**	−6.831	−6.208
**6**	−6,704	−6.308
**7**	−6.857	−6.449
**8**	−6.472	−5.993
**9**	−6.543	−6.001
**10**	−6.697	−5.799
**11**	−6.612	−6.033
**12**	−6.316	−5.520
**13**	−6.210	−5.555
**14**	−7.476	−6.759
**15**	−7.263	−6.452
**4d**	−6.394	−5.858
**5d**	−6.195	−5.990
Tariquidar	−8.273	-
Palbociclib	-	−9.011

**Fig. 8:**
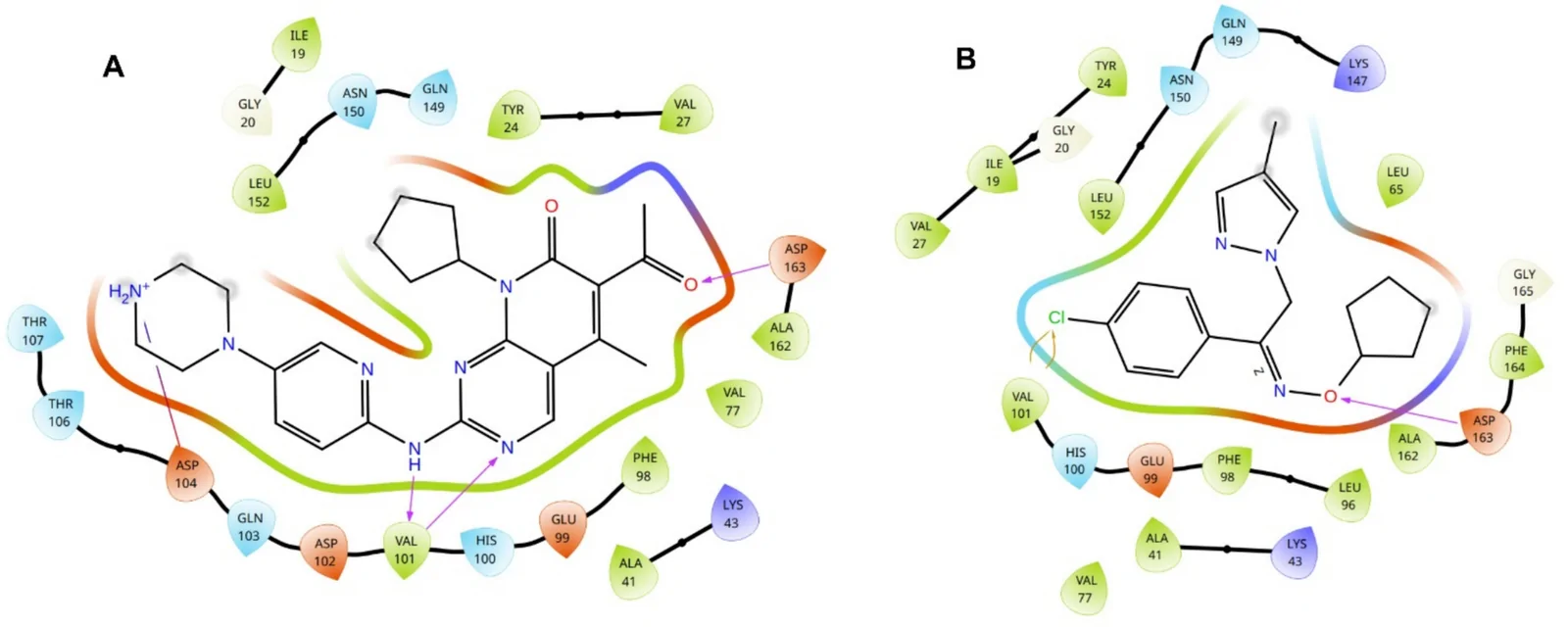
Ligand–protein 2D interactions with 5L2I **A**: Palbociclib and **B**: Compound **14**

When the 2D interactions of the compounds were evaluated in the docking results, the findings obtained were consistent with the literature data. Palbociclib formed hydrophobic interactions with Ile19, Ala41, Phe98, and Leu152 residues and polar interactions with Gln103 and Asp163 residues (Fig. 8). It was also observed that it formed hydrogen bonds with Val101, which is critically important in terms of inhibition, through the nitrogen atoms in its structure. When the interaction profile of compound **14**, which has the highest docking score, is examined (Fig. 8), it is seen that it exhibits common interactions with Palbociclib in the active site. Compound **14** formed a halogen bond with Val101 through the chlorine atom in its structure, and a hydrogen bond with Asp163, one of the key residues, through the oxygen atom of the oxime group. The intense hydrophobic contacts formed by compound **14** with Ala41, Phe98, and Leu152 residues in the hydrophobic interaction region are also noteworthy.

Palbociclib's high docking score can be explained by the density of the hydrogen bond network it forms (Bissantz et al. 2010). However, the strong hydrophobic contacts exhibited by compound **14** stand out as a decisive factor in the stable placement of the ligand in the active site and in maintaining the lipophilic character of the molecule. In a previously published study, the docking scores of compounds **4d** and **5d** were calculated as −5.858 and −5.990 kcal/mol, respectively (Alagöz et al. 2019). In this study, compound **14** (−6.759 kcal/mol), which achieved the highest docking score, surpassed both reference compounds and demonstrated that the design objective was achieved. All compounds except compounds **10**, **12** and **13** (−5.799, −5.520 and −5.555 kcal/mol, respectively) exhibited stronger interaction with the protein.

**Fig. 9:**
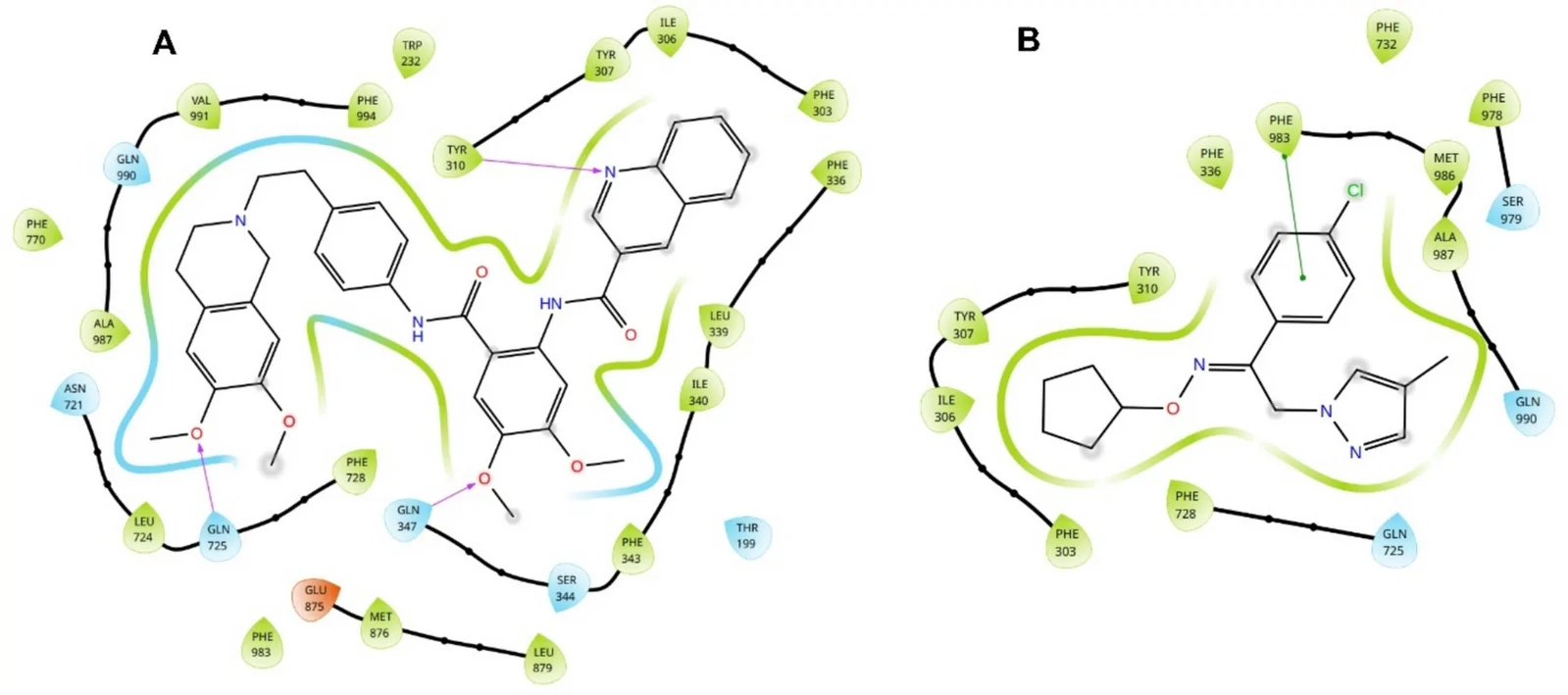
Ligand–protein 2D interactions with 7A6E **A**: Tariquidar and **B**: Compound **14**

In the docking analysis performed with P-gp (PDB: 7A6E), Tariquidar was found to be incorporated into the binding pocket in the transmembrane (TM) region by forming both hydrophobic and polar interactions, as well as hydrogen bonds. Examination of the 2D interaction profile revealed that it formed interactions compatible with residues reported in the literature to play a key role in inhibition (Fig. 9).

Hydrophobic interactions were established with Phe336, located on TM6 and playing a decisive role in inhibiting the efflux system, and with Ile340 and Phe343 residues in the same region (Nosol et al. 2020). In addition, it was observed to form hydrogen bonds with three important residues: the nitrogen atom in the quinoline ring with Tyr310, the oxygen atom in tetrahydroisoquinoline with key residue Gln347, and the oxygen atom of the other tetrahydroisoquinoline in the structure with Gln725. Thanks to this arrangement, the ligand was positioned at the correct angle and stably towards the target region of the protein. A strong cage structure is formed between the ligand and the protein through the aforementioned triple polar interaction (Nasim et al. 2020; Alam et al. 2019; Chufan et al. 2013).

The interactions of compound **14** with P-gp (7A6E) show a significant difference when compared to Tariquidar. Unlike Tariquidar, which forms polar interactions at three different key points, compound **14** is more stably located in the binding pocket through purely hydrophobic interactions (Fig. 9). This binding pattern makes it difficult for the ligand to be expelled by the protein. Hydrophobic contacts are also established with lipophilic amino acids such as Met986, Leu339, and Ile340 located around the ligand-positioned pocket. As a result, compound **14** binds to the target via strong hydrophobic contacts without the need for polar bonds and, based on *in silico* findings, stands out as a potential P-gp inhibitor candidate; this characteristic needs to be experimentally confirmed.

When the docking scores of the reference compounds **4d** and **5d** are evaluated (Alagöz et al. 2019), compound **5d** shows the weakest protein affinity in the series with −6.195 kcal/mol. Compound **4d**, with a score of −6.394 kcal/mol, lags behind only compounds **12** and **13** (−6.316 and −6.210 kcal/mol, respectively) and exhibits a lower interaction than all other new compounds. Compound **14**, with the highest binding score, shows a significantly stronger affinity with the protein compared to both **4d** and **5d**.

#### Molecular dynamics (MD) simulation

**Fig. 10:**
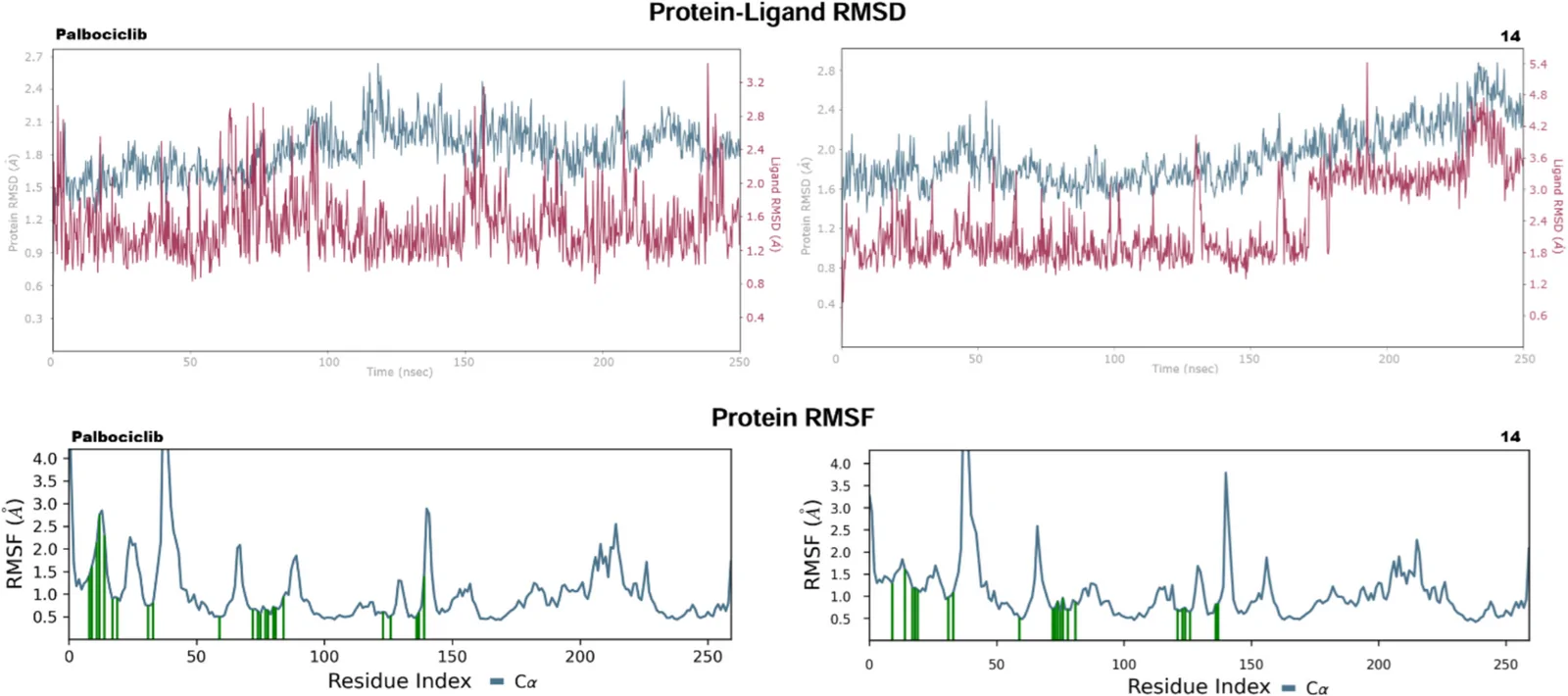
Protein–Ligand RMSD and Protein RMSF analyses resulting from MD simulations of Palbociclib and compound **14** for 250 ns

The stability of the system was investigated during MD simulations. In the Palbociclib 5L2I complex, the ligand RMSD value ranged from 0.8–3.2 Å for 250 ns, while the protein Cα RMSD value ranged from 1.2–2.6 Å. When the protein RMSF graph was evaluated (Fig. 10), it was observed that the fluctuations in the protein generally remained below 3 Å, with only limited oscillations below 4 Å observed in residues 35–45. These findings indicate that both the protein and the ligand remained largely stable during the simulation. The ligand RMSF analysis showed fluctuations in the range of 0.5–2 Å.

**Fig. 11:**
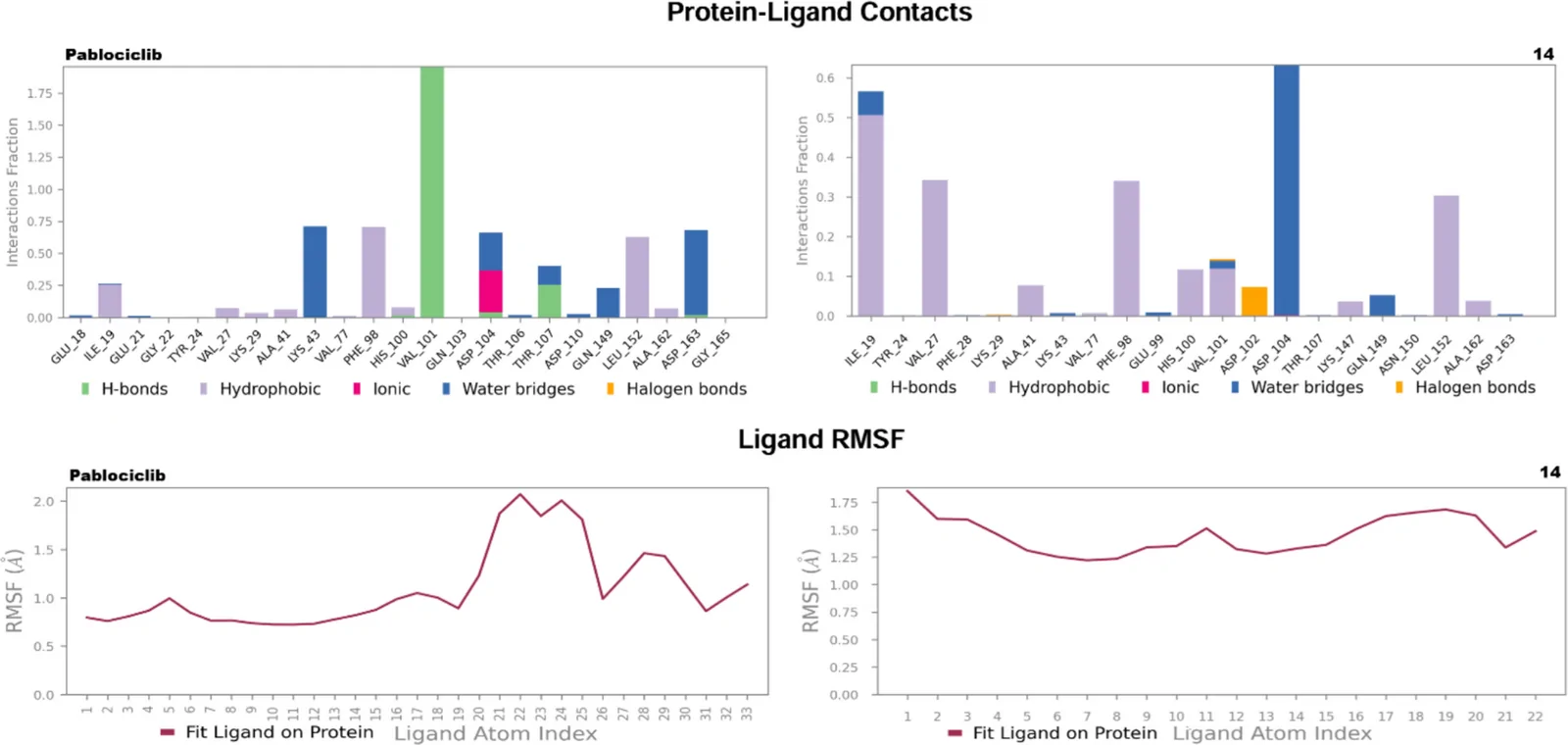
Protein–ligand contacts and ligand RMSF analyses of palbociclib and compound **14** over 250 ns MD simulations

When protein–ligand interactions were examined, it was observed that Palbociclib formed hydrogen bonds with Val101 for almost the entire duration of the simulation. Interactions with Lys43 (water bridge), Phe98 (hydrophobic), Asp104 (ionic, water bridge, hydrogen bond), Thr107 (water bridge, hydrogen bond), Leu152 (hydrophobic), and Asp163 (water bridge) were observed for approximately 40% of the simulation time. It was determined that the ligand maintained continuous intermittent interactions with Asp163, Leu152, Asp104, Val101, Phe98, and Lys43 for a large portion of the simulation time (Fig. 11).

#### ADMET analysis

Various physicochemical properties of the synthesised compounds and reference compounds were evaluated using *in silico* methods, and the results are presented in Table 4. All compounds except compounds **10** and **15** were found to be compliant with Lipinski's rule of five. Only for compounds **10** and **15**, the logP values exceed the defined limits. To evaluate the absorption and bioavailability properties of the compounds compared to reference drugs, the parameters of Percent Human Oral Absorption (PHA) and QPPCaco cell permeability (QPPCaco) were examined. The results show that all compounds have a 100% oral absorption rate and high QPPCaco values ​​in the range of 4000–6000 nm/s. Although Tariquidar (78%) and Palbociclib (76%) among the reference drugs exhibited an acceptable absorption profile, the synthesised compounds have a permeability and absorption potential exceeding these values. The fact that the oral absorption value of Doxorubicin was calculated as 0% supports the reliability and consistency of the ADME estimation method (QikProp) used.

**Table 4:** Drug potential of the designed compounds and reference compounds

Compounds	MW ≤ 500	LogP ≤ 5	HBD ≤ 5	HBA ≤ 10	Rule of Five	Percent Human Or. Abs	QPlogBB	QPPCaco	CNS	QPPMDCK	Membran dG Insert
**5**	263.726	3.33	0	4	0	100.000	0.120	4802.68	1	6660.74	1.155
**6**	277.753	3.72	0	4	0	100.000	0.105	5350.47	1	7485.56	0.441
**7**	291.780	4.14	0	4	0	100.000	0.024	5233.76	1	7309.19	−0.551
**8**	305.806	4.49	0	4	0	100.000	−0.044	5217.01	0	7283.89	−1.500
**9**	319.833	4.92	0	4	0	100.000	−0.115	5225.59	0	7296.89	−2.433
**10**	333.860	**5.32**	0	4	1	100.000	−0.169	5358.81	0	7498.14	−3.375
**11**	291.780	4.08	0	4	0	100.000	0.159	6032.71	1	8522.39	−0.169
**12**	305.805	4.43	0	4	0	100.000	0.068	5733.05	1	8065.69	−1.240
**13**	319.833	4.85	0	4	0	100.000	−0.047	5235.94	0	7312.50	−2.108
**14**	319.836	4.55	0	4	0	100.000	0.131	5755.62	1	8099.99	−0.494
**15**	345.871	**5.23**	0	4	1	100.000	0.074	5898.94	1	8318.25	−2.265
Tariquıdar	**646.74**	**5.48**	1	10	2	78.131	−0.531	326.665	1	163.317	14.360
Palbociclib	447.540	2.01	2	9	0	76.398	−0.640	127.847	1	59.247	21.586
Doxorubicin	**543.53**	−0.49	5	**12**	2	**0**	−2.998	2.272	**−2**	0.760	28.701

*MW* Molecular weight (Da), *LogP* Predicted octanol/water partition coefficient (QPlogPo/w), *HBD* Hydrogen bond donors, *HBA* Hydrogen bond acceptors; Rule of Five: Number of violations of Lipinski’s Rule of Five; Percent Human Oral Abs: Predicted human oral absorption (%); *QPlogBB* Predicted blood–brain barrier partition coefficient, *QPPCaco* Predicted Caco-2 cell permeability in nm/s, *CNS* Predicted central nervous system activity on a –2 (inactive) to + 2 (active) scale, *QPPMDCK* Predicted apparent MDCK cell permeability in nm/s

*In silico* toxicity evaluation demonstrated that none of the synthesized derivatives displayed mutagenic, tumorigenic, or reproductive toxic activity, which collectively supports their potential suitability as drug candidates from a safety perspective.

BBB penetration is a prerequisite for CNS-active therapeutic agents. In the present study, BBB permeability and CNS activity of the synthesized compounds were evaluated using the QPlogBB (acceptable range: − 3.0 to 1.2) and CNS (− 2: inactive to + 2: active) parameters. The reference compounds Doxorubicin, Tariquidar, and Palbociclib yielded CNS scores of − 2, 1, and 1, respectively, consistent with their known pharmacological profiles. Notably, all synthesized derivatives (**5–15**) exhibited QPlogBB values ranging from − 0.640 to 0.159 and CNS scores of 0–1, both within the accepted reference ranges. These findings suggest that the synthesized compounds these *in silico* parameters suggest favorable predicted BBB permeability; experimental confirmation (PAMPA-BBB/MDCK-MDR1) is required before these compounds can be considered brain-penetrant.

**Fig. 12:**
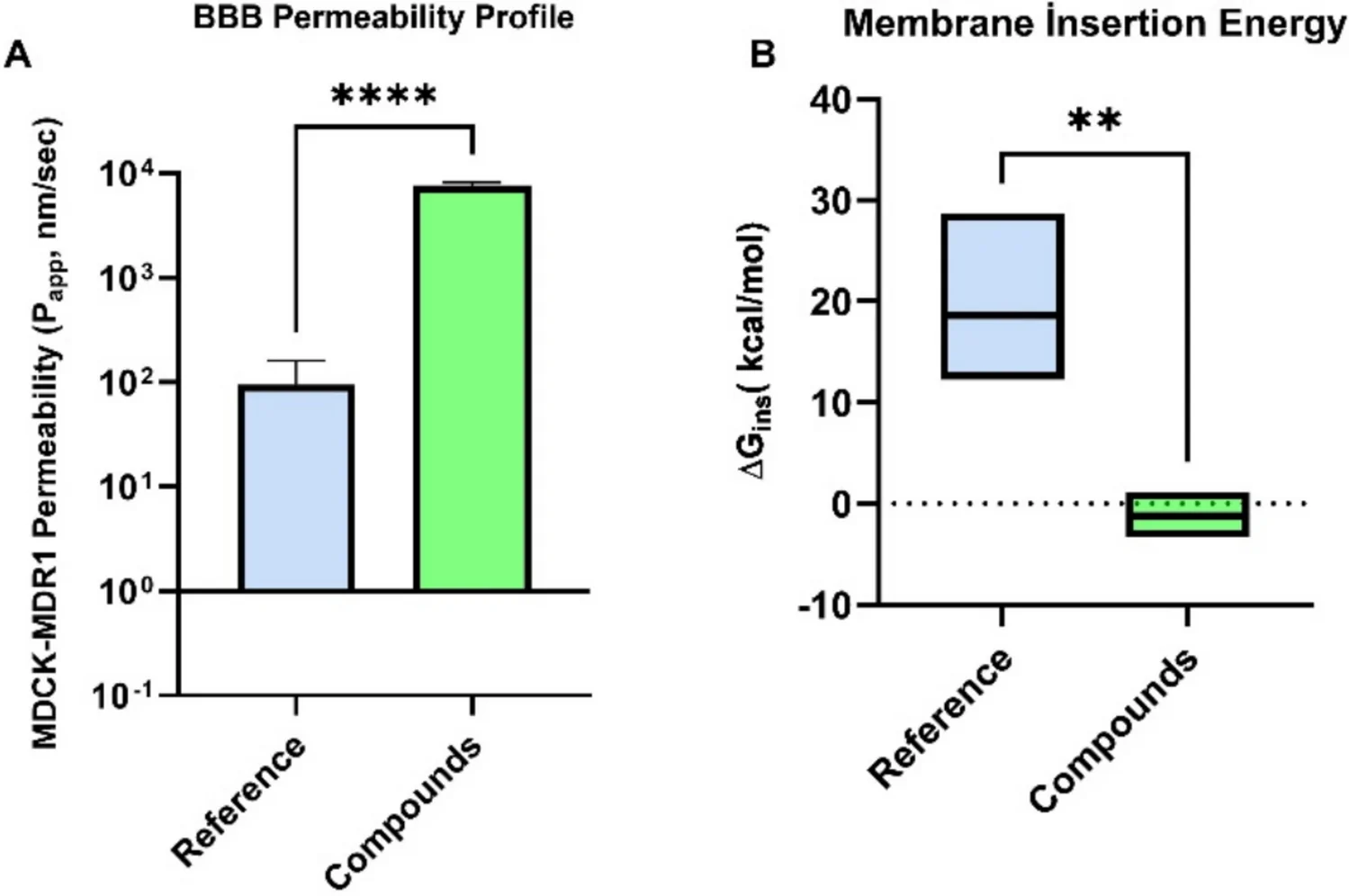
Predicted BBB transit profiles and membrane interaction energies of compounds (**5–15**) and reference drugs *in silico*. (**A**) Predicted MDCK-MDR1 (**B**) Predicted Membrane dG Insert (∆Gins), data were analyzed by Student’s t-test

The BBB penetration potential of the synthesized compounds was further evaluated through MDCK cell permeability and membrane insertion energy analyses, and the results are presented in Fig. 12. Examination of the data revealed that Doxorubicin, Tariquidar, and Palbociclib exhibited a low mean permeability of approximately 74 nm/sec, whereas the synthesized compounds demonstrated a considerably higher permeability profile, with a mean value of 7623 nm/sec. This marked difference indicates that the synthesized derivatives are capable of traversing biological membranes via passive diffusion more effectively than the reference compounds. When the Membrane dG (insert) values, which reflect the thermodynamic tendency of a molecule to insert into the membrane, were assessed, the synthesized compounds displayed a mean value of approximately − 1.14, with a near-zero negative tendency, while this value was found to be markedly positive at + 21.5 for the reference compounds (Fig. 12). A negative or near-zero Membrane dG (insert) value indicates that membrane penetration is not an energetically forced process but rather one that tends to occur spontaneously under thermodynamic conditions. This finding suggests that the synthesized compounds are able to progress toward intracellular targets without encountering a significant energy barrier. Furthermore, when dipole moment values were compared, the synthesized derivatives exhibited a lower mean polarity of 3.6 Debye relative to the reference drugs (5.1 Debye). Reduced polarity is one of the key determinants that facilitates passage across lipophilic cell membranes. The BBB permeability predictions reported herein are based solely on in silico calculations (QikProp, Schrödinger). Although the predicted QPPMDCK and QPlogBB values are consistent with established reference ranges for CNS-active compounds, experimental validation using PAMPA-BBB or MDCK-MDR1 permeability assays was not performed in the current study. These in silico estimates should therefore be interpreted as preliminary indicators of CNS penetration potential, and experimental confirmation is warranted in future studies.

#### SAR analysis

The SAR of the designed compound series was evaluated based on the effects of structural modifications at the R position alkyl chains on physicochemical properties and *in vitro*/*in silico* activities. Elongation of the alkyl chain from C1 to C6 and the corresponding increase in carbon number predictably enhanced the lipophilic character of the molecules, resulting in a progressive increase in LogP values from 3.33 to 5.32 (Table 4). However, the linear correlation between LogP increase and cytotoxic activity was limited to chains of up to three carbons (methyl, ethyl, and propyl), as evidenced by the C6 cell line IC₅₀ values of 41.18, 29.74, and 22.41 µM, respectively (Table 2). Molecular docking studies confirmed that this relationship stemmed not merely from a physicochemical trend but rather from differences in pharmacodynamic interactions within the binding pocket. To investigate the effect of alkyl chain branching on biological activity, the straight-chain (*n-*) and branched (*iso-*) isomers of propyl, butyl, and pentyl groups were comparatively synthesized. *In vitro* findings demonstrated that the transition to iso- derivatives markedly attenuated antiproliferative activity against the C6 cell line (Table 2). This decline in activity directly correlated with the reduced docking scores, which indicated a corresponding decrease in affinity toward the target proteins (Table 3). To determine the effect of cyclic structures on protein binding, the *n-*, *iso-*, and cyclo- forms of the pentyl derivative were specifically analyzed. In silico data clearly demonstrated that the cyclo- form exhibited the highest binding affinity toward both target proteins, whereas the *iso-* form displayed the weakest interactions, as reflected by the docking scores against 7A6E (− 6.543, − 6.210, and − 7.263 kcal/mol, respectively) and 5L2I (− 6.001, − 5.555, and − 6.759 kcal/mol, respectively). When the relationship between in silico predictions and *in vitro* findings was examined, these data were found to be in good agreement with the IC₅₀ results for the *n-*, *iso-*, and cyclo- derivatives (59.37, 139.95 ve 31.23 µM, respectively). The fact that the compounds bearing cyclopentyl and methylcyclohexyl substituents achieved the highest docking scores across the entire series (Table 3) the docking results are consistent with cyclic substituents engaging the binding pocket more favorably than linear/branched chains.

#### Computational NMR calculations and DP4 + analysis

Compounds **7** and **9** were obtained as *E/Z* mixtures, while the remaining compounds were obtained as single isomers. Since the compounds could not be obtained in crystalline form, the structures were determined using spectroscopic methods, namely ^13^C-NMR, ^1^H-NMR, and HRMS. Computational NMR/DP4 + analysis was carried out on compounds **5** and **9** as representative examples in order to identify the *E/Z* isomers. This analysis showed that compound **5** is 100% the *E* isomer. For compound **9**, the analysis was performed using the sigma values of the major isomer signals in the NMR data, and the major isomer was determined to be 80% *E*. The analysis results supporting these findings have been added to the supporting information file. Given the results obtained for compounds **5** and **9**, together with the fact that the other compounds were synthesized by the same method under the same conditions and share structural similarity, we conclude that these compounds are likewise the *E* isomer.

## Materials and methods

### Chemistry

#### General

All chemicals used in this study were purchased from Merck and Sigma-Aldrich. Merck Kieselgel 60 plates were employed for thin-layer chromatography (TLC) to monitor reaction progress, as well as for preparative TLC procedures used in the purification of the synthesized compounds. For structural elucidation, ^1^H-NMR spectra were recorded on a Bruker Avance 600 MHz Ultrashield™ NMR spectrometer and ^13^C-NMR spectra were acquired on a Bruker Avance 150 MHz Ultrashield™ NMR spectrometer. Deuterated chloroform (CDCl₃, Merck) was used as the solvent for all spectrometric analyses. Splitting patterns are reported as follows: s (singlet), d (doublet), t (triplet), q (quartet), and m (multiplet). High-resolution mass spectra (HRMS) of the synthesized compounds were recorded using a Thermo Scientific Q Exactive LC–MS/MS system equipped with an electrospray ionization (ESI) source operating in positive ion mode. Data acquisition and processing were performed using Xcalibur software.

#### General synthesis procedure for intermediate products

##### Synthesis of 1-(4-Chlorophenyl)−2-(1***H***−4-methylpyrazole-1-yl)ethane

0.03 mol of 4-methylpyrazole was dissolved in 2.5 mL of dimethylformamide (DMF), and the resulting solution was cooled to 0 °C in an ice bath. A separately prepared solution of 0.01 mol of 4′-chloro-2-bromoacetophenone in 2.5 mL of DMF was added dropwise to the cooled 4-methylpyrazole solution with continuous stirring on a magnetic stirrer. The reaction mixture was maintained in the ice bath for 2 h and subsequently allowed to stir at room temperature for 1 day. Upon completion of the reaction, the mixture was poured dropwise into ice-cold water. The resulting precipitate was collected by filtration, washed with distilled water, and dried. The product was obtained in pure form (Alagöz et al. 2019).

##### Synthesis of 1-(4-Chlorophenyl)−2-(1***H***−4-methylpyrazole-1-yl)ethanone oxime

0.015 mol of 1-(4-chlorophenyl)−2-(4-methyl-1*H*-pyrazol-1-yl)ethanone was dissolved in 75 mL of ethanol and heated under reflux with continuous stirring. Subsequently, 0.03 mol of hydroxylamine hydrochloride was added to the reaction mixture. The reaction medium was basified to pH 8 by the addition of 15 N sodium hydroxide solution, and the mixture was stirred under reflux for 1–2 h. The reaction mixture was then concentrated under reduced pressure using a rotary evaporator until complete removal of ethanol. Ice-cold water was added to the resulting solid residue, and the mixture was stirred on a magnetic stirrer for 4 h. The product was finally collected by filtration and obtained in pure form (Alagöz et al. 2019).

#### General procedure for target compounds (5–15)

##### (*E*)- 1-(4-Chlorophenyl)−2-(4-methyl-1*H*-pyrazol-1-yl)ethanone O-methyl oxime (5)

Yield: %68; white powder. ^1^H-NMR (600 MHz, CDCl_3_-d): δ 1.94 (3H; s, pyrazole-CH_3_), 4.00 (3H; s, O-CH_3_), 5.23 (2H; s, C-CH_2_-N), 7.11 (H; s, = CH; pyrazole), 7.19 (H; s, = CH; pyrazole), 7.21–7.23 (2H; m, = CH; phenyl), 7.58–7.60 (2H; m, = CH; phenyl). ^13^C-NMR (150 MHz, CDCl_3_-d): δ 150.57, 138.71, 134.54, 131.33, 127.68, 127.43, 126.84, 115.74, 61.65, 44.63, 7.85. HRMS (ESI): *m/z* calcd for C₁₃H₁₄ClN₃O [M + H]⁺ 264.08982; found: 264.08984.

##### (*E*)−1-(4-Chlorophenyl)−2-(4-methyl-1*H*-pyrazol-1-yl)ethanone O-ethyl oxime (6)

Yield: %74; White powder. ^1^H-NMR (600 MHz, CDCl_3_-d): δ 1.29 (3H; t, -CH_2_-CH_3_), 1.94 (3H; s, pyrazole -CH_3_), 4.26 (2H; q, O-CH_2_), 5.25 (2H; s, C-CH_2_-N), 7.12 (H; s, = CH; pyrazole), 7.19 (H; s, = CH; pyrazole), 7.21–7.23 (2H; m, = CH; phenyl), 7.59–7.60 (2H; m, = CH; phenyl). ^13^C-NMR (150 MHz, CDCl_3_-d): δ 150.19, 138.62, 134.40, 131.60, 127.66, 127.44, 126.79, 115.66, 69.61, 44.75, 13.70, 7.86. HRMS (ESI): *m/z* calcd for C₁₄H₁₆ClN₃O [M + H]⁺ 278.10547; found: 278.10547.

##### (***E/Z***)−1-(4-Chlorophenyl)−2-(4-methyl-1***H***-pyrazole-1-yl)ethanone O-propyl oxime (7)

Yield: %71.25; white powder. ^1^H-NMR (600 MHz, CDCl_3_-d): δ: 0.92 (3H; t, -C_2_H_4_-CH_3_), 1.71 (2H, m, CH_2_-CH_3_), 1.94 (3H; s, pyrazole-CH_3_), 4.17 (2H; t, O-CH_2_), 5.26 (2H; s, C-CH_2_-N), 7.12 (H; s, = CH; pyrazole), 7.18 (H; s, = CH; pyrazole), 7.27–7.29 (2H; m, = CH; phenyl), 7.59–7.60 (2H; m, = CH; phenyl). ^13^C-NMR (150 MHz, CDCl_3_-d): δ 149.37, 138.60, 134.15, 128.80, 127.67, 127.39, 126.78, 115.6, 54.37, 44.73, 30.91, 21.27, 13.10, 7.87. HRMS (ESI): *m/z* calcd for C₁₅H₁₈ClN₃O [M + H]⁺ 292.12112; found: 292.12112.

##### (***E***)−1-(4-Chlorophenyl)−2-(4-methyl-1***H***-pyrazole-1-yl)ethanone O-butyl oxime (8)

Yield: %80; white powder. ^1^H-NMR (600 MHz, CDCl_3_-d): δ 0.90 (3H; t, -C_3_H_6_-CH_3_), 1.36 (2H, m, CH_2_-CH_3_), 1.67 (2H, m, CH_2_-CH_2_), 1.95 (3H; s, pyrazole-CH_3_), 4.21(2H; t, O-CH_2_), 5.25 (2H; s, C-CH_2_-N), 7.12 (H; s, = CH; pyrazole), 7.19 (H; s, = CH; pyrazole), 7.22–7.23 (2H; m, = CH; phenyl), 7.59–7.61 (2H; m, = CH; phenyl). ^13^C-NMR (150 MHz, CDCl_3_-d): δ 138.59, 134.40, 131.59, 127.68, 127.47, 126.78, 115.67, 73.93, 44.77, 30.21, 28.68, 18.14, 12.87, 7.85. HRMS (ESI): *m/z* calcd for C₁₆H₂₀ClN₃O [M + H]⁺ 306.13677; found: 306.13693.

##### (***E/Z***)−1-(4-Chlorophenyl)−2-(4-methyl-1***H***-pyrazole-1-yl)ethanone O-pentyl oxime (9)

Yield: %73; white powder. ^1^H-NMR (600 MHz, CDCl_3_-d): δ 0.85 (3H; t, -C_4_H_8_-CH_3_), 1.30 (2H, m, CH_2_-CH_3_), 1.32 (2H, m, CH_2_-CH_2_), 1.68 (2H, m, CH_2_-CH_2_), 1.94 (3H; s, pyrazole-CH_3_), 4.20 (2H; t, O-CH_2_), 5.24 (2H; s, C-CH_2_-N), 7.12 (H; s, = CH; pyrazole), 7.19 (H; s, = CH; pyrazole), 7.22–7.23 (2H; m, = CH; phenyl), 7.59–7.61 (2H; m, = CH; phenyl). ^13^C-NMR (150 MHz, CDCl_3_-d): δ 150.08, 138.62, 134.39, 131.64, 128.81, 127.67, 126.78, 115.64, 74.22, 54.40, 44.78, 27.12, 21.46, 13.01, 7.85. HRMS (ESI): *m/z* calcd for C₁₇H₂₂ClN₃O [M + H]⁺ 320.15242; found: 320.15244.

##### (***E***)−1-(4-Chlorophenyl)−2-(4-methyl-1***H***-pyrazole-1-yl)ethanone O-hexyl oxime (10)

Yield: %60; white powder. ^1^H-NMR (600 MHz, CDCl_3_-d): δ 0.83 (3H; t, -C_5_H_10_-CH_3_), 1.18 (2H; m, CH_2_-CH_3_), 1.26 (2H; m, CH_2_-CH_2_), 1.33 (2H; m, CH_2_-CH_2_), 1.67 (2H; m, CH_2_-CH_2_), 1.94 (3H; s, pyrazole-CH_3_), 4.20 (2H; t, O-CH_2_), 5.24 (2H; s, C-CH_2_-N), 7.12 (H; s, = CH; pyrazole), 7.19 (H; s, = CH; pyrazole), 7.22–7.23 (2H; m, = CH; phenyl), 7.59–7.61 (2H; m, = CH; phenyl). ^13^C-NMR (150 MHz, CDCl_3_-d): δ 150.07, 138.62, 134.39, 131.64, 127.66, 127.45, 126.78, 115.64, 74.23, 44.76, 30.59, 28.11, 24.62, 21.59, 13.00, 7.84. HRMS (ESI): *m/z* calcd for C₁₈H₂₄ClN₃O [M + H]⁺ 334.16807; found: 334.16818.

##### (***E***)−1-(4-Chlorophenyl)−2-(4-methyl-1***H***-pyrazole-1-yl)ethanone O-isopropyl oxime (11)

Yield: %70; white powder. ^1^H-NMR (600 MHz, CDCl_3_-d): δ 1.275 (6H; d, -CH-(CH_3_)_2_), 1.95 (3H; s, pyrazole-CH_3_), 4.48 (H; m, O–CH), 5.24 (2H; s, C-CH_2_-N), 7.13 (H; s, = CH; pyrazole), 7.19 (H; s, = CH; pyrazole), 7.21–7.23 (2H; m, = CH; phenyl), 7.61–7.62 (2H; m, = CH; phenyl). ^13^C-NMR (150 MHz, CDCl_3_-d): δ 149.64, 138.52, 134.27, 131.86, 127.63, 127.47, 126.73, 115.58, 54.51, 44.78, 20.71, 7.87. HRMS (ESI): *m/z* calcd for C₁₅H₁₈ClN₃O [M + H]⁺ 292.12112; found: 292.12112.

##### (***E***)−1-(4-Chlorophenyl)−2-(4-methyl-1***H***-pyrazole-1-yl)ethanone O-isobutyl oxime (12)

Yield: %77; white powder. ^1^H-NMR (600 MHz, CDCl_3_-d): δ 0.91 (6H; d, -CH(CH_3_)_2_), 1.94 (3H; s, pyrazole-CH_3_), 2.02 (1H; m, -CH(CH_3_)_2_), 3.99 (2H; t, O-CH_2_), 5.26 (2H; s, C-CH_2_-N), 7.12 (H; s, = CH; pyrazole), 7.19 (H; s, = CH; pyrazole), 7.22–7.23 (2H; m, = CH; phenyl), 7.59–7.60 (2H; m, = CH; phenyl). ^13^C-NMR (150 MHz, CDCl_3_-d): δ 150.03, 138.62, 134.40, 131.61, 127.67, 127.46, 126.78, 115.68, 80.65, 44.78, 27.09, 18.16, 7.84. HRMS (ESI): *m/z* calcd for C₁₆H₂₀ClN₃O [M + H]⁺ 306.13677; found: 306.13681.

##### (***E***)−1-(4-Chlorophenyl)−2-(4-methyl-1***H***-pyrazole-1-yl)ethanone O-isopentyl oxime (13)

Yield: %74; white powder. ^1^H-NMR (600 MHz, CDCl_3_-d): δ 0.89 (6H; d, -CH-(CH_3_)_2_), 1.58 (2H; m, -CH_2_-CH_2_-CH-), 1.66 (1H; m, CH-(CH_3_)_2_), 1.94 (3H; s, pyrazole-CH_3_), 4.24 (2H; t, O-CH_2_), 5.24 (2H; s, C-CH_2_-N), 7.12 (H; s, = CH; pyrazole), 7.19 (H; s, = CH; pyrazole), 7.22–7.23 (2H; m, = CH; phenyl), 7.59–7.60 (2H; m, = CH; phenyl). ^13^C-NMR (150 MHz, CDCl_3_-d): δ 150.08, 138.62, 134.40, 131.61, 127.67, 127.46, 126.78, 115.67, 44.78, 36.89, 28.68, 24.07, 21.61, 7.85. HRMS (ESI): *m/z* calcd for C₁₇H₂₂ClN₃O [M + H]⁺ 320.15242; found: 320.15247.

##### (***E***)−1-(4-Chlorophenyl)−2-(4-methyl-1***H***-pyrazole-1-yl)ethanone O-cyclopentyl oxime (14)

Yield: % 68; white powder. ^1^H-NMR (600 MHz, CDCl_3_-d): δ 1.61 (4H; m, cyclopentyl), 1.82 (4H; m, cyclopentyl), 1.94 (3H; s, pyrazole-CH_3_), 4.81 (1H; m, O–CH-), 5.21 (2H; s, C-CH_2_-N), 7.10 (H; s, = CH; pyrazole), 7.19 (H; s, = CH; pyrazole), 7.22–7.23 (2H; m, = CH; phenyl), 7.61–7.62 (2H; m, = CH; phenyl). ^13^C-NMR (150 MHz, CDCl_3_-d): δ 150.01, 138.55, 134.30, 131.87, 127.65, 127.44, 126.77, 115.55, 85.50, 44.85, 31.18, 22.76, 7.85. HRMS (ESI): *m/z* calcd for C₁₇H₂₂ClN₃O [M + H]⁺ 320.15242; found: 320.15244.

##### (***E***)−1-(4-Chlorophenyl)−2-(4-methyl-1***H***-pyrazole-1-yl)ethanone O-cyclohexylmethyl oxime (15)

Yield: %55; white powder. ^1^H-NMR (600 MHz, CDCl_3_-d): δ 0.93 (2H; m, cyclohexyl), 1.21 (1H; m, -CH_2_-CH-), 1.63 (2H; m, cyclohexyl), 1.71 (4H; m, cyclohexyl), 1.94 (3H; s, pyrazole-CH_3_), 4.02 (2H; t, O-CH_2_), 5.24 (2H; s, C-CH_2_-N), 7.12 (H; s, = CH; pyrazole), 7.19 (H; s, = CH; pyrazole), 7.21–7.23 (2H; m, = CH; phenyl), 7.59–7.60 (2H; m, = CH; phenyl). ^13^C-NMR (150 MHz, CDCl_3_-d): δ 149.94, 138.61, 134.37, 131.64, 127.66, 127.48, 126.77, 115.63, 79.72, 44.79, 36.60, 28.79, 25.48, 24.75, 7.84. HRMS (ESI): *m/z* calcd for C₁₉H₂₄ClN₃O [M + H]⁺ 346.16807; found: 346.16803.

### Biological activity studies

#### Cell culture and MTT assay

This study was conducted on the MCF-7 (human breast adenocarcinoma) and C6 (rat glioma) cell lines. These cell lines were selected to investigate the effects of the synthesized compounds on breast cancer and to evaluate their cytotoxic effects on brain tumors, which frequently metastasize. The SH-SY5Y human neuroblastoma cell line (ATCC® CRL-2266) was included as a neuronal reference line to assess the selectivity of the synthesized compounds toward cancer cells relative to CNS-derived cells. Although SH-SY5Y cells are derived from a pediatric neuroblastoma and do not represent primary neurons, they are widely employed as a neuronal surrogate model in CNS-targeted drug discovery studies (Kovalevich and Langford 2013).

MCF-7, C6, and SH-SY5Y cell lines were thawed, cultured, and cryopreserved for experimental use. Frozen cells (~ 1 mL) were rapidly warmed in a 37 °C water bath, seeded evenly into 25 cm^2^ flasks, and incubated for 48 h at 37 °C in a humidified atmosphere with 5% CO₂ to allow proliferation. Cells were maintained in Dulbecco’s Modified Eagle Medium (DMEM) supplemented with 10% fetal bovine serum (FBS) and 1% penicillin/streptomycin. When cultures reached confluence, cells were washed with PBS, detached using trypsin, and either replated for continued culture or suspended in DMSO and stored in cryovials at −20 °C briefly, followed by long-term storage at −80 °C.

##### MTT assays

The MTT [3-(4,5-dimethylthiazol-2-yl)−2,5-diphenyltetrazolium bromide] assay was performed to determine the IC₅₀ of the compound, which represents the concentration required to inhibit 50% of cell viability. Cells were plated in sterile 96-well plates at a density of 1 × 10^5^ cells per well, with cell counts confirmed using an Olympus R1 cell counter. The compound was dissolved in DMSO, and serial dilutions (100.0, 50.0, 25.0, 12.5, 6.25, and 3.125 μg/mL) were prepared, with 2 μL of each concentration added to the respective wells. Following 24 h of incubation, 20 μL of MTT solution (5 mg/mL in PBS) was added to each well. After 2 h of further incubation, the medium was removed, and 100 μL of DMSO was added to solubilize the formed formazan crystals. Absorbance was measured at 540 nm using a microplate reader (Thermo Fisher Scientific, USA). The cell viability and IC50 value were calculated using the SigmaPlot 12.0 program (Sudhadevi et al. 2024).

$$\mathrm{Cell}\;\mathrm{Viability}\;\%=\left({\mathrm A}_{\mathrm{sample}}-{\mathrm A}_{\mathrm{blank}}\right)/\left({\mathrm A}_{\mathrm{standart}}-{\mathrm A}_{\mathrm{blank}}\right)\;\mathrm x\;100$$

#### Flow cytometry

Apoptosis was assessed in C6 and SH-SY5Y cell lines using the Annexin V-fluorescein isothiocyanate (FITC)/PI assay to quantify the ratio of apoptotic to necrotic cells. Cells were treated with the IC₅₀ concentration of the compound and incubated for 24 h. After incubation, cells were trypsinized, centrifuged at 800 rpm for 8 min, and washed with cold phosphate-buffered saline (PBS). The cell pellet was resuspended in 100 μL of binding buffer and stained with 5 μL each of Annexin V-FITC and PI. Samples were incubated for 15 min at room temperature in the dark, followed by the addition of 400 μL of binding buffer. Apoptosis was analyzed according to the manufacturer’s instructions (excitation: 488 nm; emission: 530 nm) (Topçu et al. 2024).

#### Cell cycle analysis

Cell cycle analysis was performed on the C6 cell line to identify the phase at which the compound induces cell cycle arrest, using the Muse™ Cell Cycle Kit (Cat. No. MCH100106) according to the manufacturer’s instructions. Cells were treated with the IC₅₀ concentration and incubated for 24 h. After incubation, cells were trypsinized, centrifuged at 800 rpm for 8 min, and washed with cold PBS. A volume of 200 μL of cell suspension (1 × 10⁶ cells/mL) was transferred to each tube, centrifuged at 300 × g for 5 min, and washed once with PBS. While gently mixing, 200 μL of ice-cold 70% ethanol was added dropwise, and cells were incubated at −20 °C for at least 3 h. Cells were then centrifuged at 300 × g for 5 min, washed with PBS, and stained with 200 μL of Muse™ Cell Cycle Reagent. Samples were incubated in the dark at room temperature for 30 min prior to analysis (Topçu et al. 2024).

Cells were fixed with 100% ethanol (C6). A DAPI solution was prepared at a concentration of 5 mg/mL in water. After fixation, the cells were washed three times with PBS. A 300 nM DAPI staining solution was added to cover the cells. The staining solution was then removed, and the cells were washed three times with PBS. Images were captured using an Olympus microscope with excitation/emission wavelengths of 358/461 nm (Güleç et al. 2024).

### *In silico* studies

All *in silico* investigations were performed utilizing the Maestro software suite (Schrödinger LLC, New York, release 2025–1). The crystal structures of the target proteins, specifically 5L2I (human CDK6 co-crystallized with Palbociclib) and 7A6E (human ABCB1 in complex with MRK16 Fab and Tariquidar), were retrieved from the RCSB Protein Data Bank (PDB) (Chen et al. 2016; Nosol et al. 2020). Protein preparation was executed via the Protein Preparation Wizard module; this involved the assignment of bond orders, addition of missing hydrogen atoms, determination of protonation states at physiological pH, and the removal of crystallographic water molecules where appropriate. Subsequent energy minimization was conducted employing the OPLS4 force field. The target ligands were initially drawn using the 2D Sketcher tool in Maestro. Their corresponding protonation states, tautomers, and low-energy conformations were generated using the LigPrep module. During this preparation phase, the Epik tool was utilized to predict possible protonation and tautomeric states within a pH range of 7.0 ± 2.0, permitting the net charge of each ligand to adjust according to the specified conditions. Finally, the ligand geometries were optimized applying the OPLS4 force field (Adal et al. 2025; Zoatier et al. 2025).

#### Molecular docking

A receptor grid box with dimensions of 20 × 20 × 20 Å was generated around the center of the binding pocket, defined by the coordinates of the native ligands present in the protein structures. The prepared ligands were subsequently docked into these specified grid centers utilizing the Glide Standard Precision (SP) protocol, configured to generate 50 poses per ligand. Finally, the intermolecular interactions of the resulting poses with key active site residues were analyzed via the Schrödinger XP Visualizer, and the docking scores were reported in kcal/mol (Adal et al. 2025; Zoatier et al. 2025).

#### Molecular dynamics simulation studies

Molecular dynamics (MD) simulations were performed in the Desmond module to validate the molecular docking results and to assess the stability of the ligands within the active site of the target protein. In these simulations, the behavior of compound **14** and Palbociclib in the 5L2I structure was evaluated to be over 250 ns under the NPT ensemble. The systems were solvated in an octahedral box using TIP3P water molecules, maintaining a minimum solute–box distance of 10 Å. Na⁺ and Cl⁻ ions were added to neutralize the systems and to achieve a physiological ionic strength (0.15 M NaCl). Desmond’s default relaxation protocol was applied, and prior to production runs, the solvated systems were subjected to sequential energy minimizations followed by short MD equilibration steps. Temperature was maintained at 300 K using the Nosé–Hoover chain thermostat, and pressure was controlled at ~ 1.01325 bar using the Martyna–Tobias–Klein barostat with isotropic coupling. Short-range nonbonded interactions were treated with a 9 Å cutoff, and long-range electrostatics were computed using the particle mesh Ewald (PME) method (Alagoz et al. 2022; Desai et al. 2024).

#### ADMET analysis

Prior to the *in silico* evaluations, both the synthesized and reference compounds were structurally prepared using LigPrep, followed by energy minimization employing the OPLS4 force field within the MacroModel program (Barut et al. 2021). Subsequently, various physicochemical parameters (MW, LogP, HBD, HBA, Rule of Five, Percent Human Oral Abs., QPlogBB, QPPCaco, CNS, QPPMDCK membrane dG insert, dipole moment value) evaluated.

The toxicity risk profiles of the synthesized compounds, including mutagenicity, tumorigenicity and reproductive effects, were assessed using the DataWarrior software (v6.1) based on structural fragment analysis derived from the RTECS database (Sander et al. 2015; Lopez-Lopez et al. 2019).

#### Computational NMR calculations and DP4 + analysis

To determine the relative configuration of the *E* and *Z* isomers of compounds **5** and **9**, DFT-NMR calculations were performed and DP4 + statistical analysis was applied. Both candidate structures (the *E* and *Z* isomers) were first pre-optimized using a molecular mechanics minimization step. Full geometry optimization was then carried out at the B3LYP-D3/6-31G** level of theory with the PCM solvent model (chloroform) using Jaguar software (Schrödinger LLC, New York, release 2025–1). The calculated isotropic shielding values were compared with the experimental ^1^H and ^13^C NMR chemical shifts using the DP4 + App software (v2.2.1). Based on Bayes theorem, the DP4 + analysis used the ^1^H and ^13^C chemical shift data to evaluate the probability that each candidate structure (*E* or *Z*) matched the experimental data for compounds **5** and **9**. (Zanardi and Sarotti 2021; Grimblat et al. 2015).

## Conclusions

In this study, eleven novel hybrid compounds (**5–15**) incorporating oxime ether derivatives of the 4-methylpyrazole core were designed, synthesized, and evaluated through comprehensive biological and *in silico* methods. These hybrid compounds, developed by integrating pyrazole and oxime ether scaffolds, were aimed at investigating dual-target cytotoxic activity against both glioma and breast cancer cell lines with brain metastatic potential. *In vitro* cytotoxicity assays demonstrated that the synthesized derivatives exhibited significantly more potent and selective antiproliferative activity against the C6 glioma cell line compared to MCF-7 breast cancer cells. Compound **14**, which possessed the highest selectivity index within the entire series (SI C6 = 9.02; C6 IC₅₀ = 31.23 µM), emerged as the most promising candidate and displayed no appreciable cytotoxicity against the SH-SY5Y neuronal reference cell line. Although compound **7** yielded the lowest IC₅₀ value within the series (C6: 22.41 µM), its selectivity index was considerably lower than that of compound **14**. Mechanistic studies conducted on compound **14** revealed that its cytotoxic effect is mediated through apoptosis induction and cell cycle arrest. Flow cytometry analyses demonstrated a dose-dependent increase in early and late apoptotic populations in C6 cells. Cell cycle analysis further indicated that compound **14** triggered cell accumulation at the G2/M checkpoint, thereby preventing mitotic entry. DAPI nuclear staining visually confirmed apoptosis-associated morphological alterations, including nuclear fragmentation and chromatin condensation. Molecular docking predicted that compound **14** interacts with key residues overlapping with the reference compound palbociclib in the active site of CDK6 (PDB: 5L2I). In the transmembrane binding pocket of P-glycoprotein (PDB: 7A6E), the compound demonstrated stable accommodation exclusively through hydrophobic contacts at the same region occupied by Tariquidar. MD simulations indicated that the predicted binding pose of compound **14** in CDK6 was maintained over the trajectory. *In silico* ADMET studies revealed that the majority of the synthesized compounds complied with Lipinski's rule of five, all exhibited 100% oral absorption potential, and possessed a physicochemical profile conducive to passive diffusion across the blood–brain barrier, as evidenced by the calculated QPPMDCK and QPlogBB values. SAR analyses demonstrated that the length and branching pattern of the alkyl chain attached to the oxygen atom of the oxime ether play a decisive role in both cytotoxic activity and target protein affinity. Among the linear-chain derivatives, increasing lipophilicity from C1 to C3 correlated positively with biological activity; however, this relationship was disrupted for chains of C4 and beyond. Branched isoforms exhibited a marked loss of activity, whereas cyclic structures were found to engage more favorably with both target proteins compared to their linear or branched counterparts. The findings suggest that the 4-methylpyrazole–oxime ether hybrid scaffold offers a promising platform for the design of glioma and brain metastasis-targeted therapeutics. Compound **14**, with its predicted interactions with CDK6 and P-glycoprotein and its favorable *in silico* physicochemical profile, represents a particularly noteworthy candidate warranting further investigation.

## Supplementary Information

Below is the link to the electronic supplementary material.

Supplementary file1 (DOC 1220 KB)

## Data Availability

All source data for this work (or generated in this study) are available upon reasonable request.
