# Supplementary material for: Rational design of pyrazole–oxime hybrids as dual-targeting agents for glioma and breast cancer brain metastasis
Source: Naunyn Schmiedebergs Arch Pharmacol. 2026 Jun 27;399(12):19685–707. doi: 10.1007/s00210-026-05618-w (PMC13593641; doi:10.1007/s00210-026-05618-w)
Supplement: Supplementary file 1 [file 210_2026_5618_MOESM1_ESM.doc]

**SUPPLEMENTARY INFORMATION**

**Rational Design of Pyrazole–Oxime Hybrids as Dual-Target Anticancer Agents for Glioma and Breast Cancer Brain Metastases**

Merve Gul^1^, Mehmet Abdullah Alagoz^1,*^, Ceylan Hepokur^2^, Oztekin Algul^3,4^

^1^Department of Pharmaceutical Chemistry, Faculty of Pharmacy, Inonu University, Malatya, Türkiye

^2^Department of Biochemistry, Faculty of Pharmacy, Sivas Cumhuriyet University, Sivas, Türkiye

^3^Department of Pharmaceutical Chemistry, Faculty of Pharmacy, Mersin University, Mersin, Türkiye

^4^Department of Pharmaceutical Chemistry, Faculty of Pharmacy, Erzincan Binali Yıldırım University, Erzincan, Türkiye

**Correspondence:** Mehmet Abdullah Alagoz ([mehmet.alagoz@inonu.edu.tr](mailto:mehmet.alagoz@inonu.edu.tr))

**Running title**; Novel Pyrazole–Oxime Hybrids as Anticancer Agents

| **Table of Contents** | **Page** |
| --- | --- |
| The synthesized compounds | 2 |
| ^1^H, ^13^C NMR and Mass spectra of the synthesized compounds (**5-15**) | 3-19 |
| Computational NMR calculations and DP4+ analysis compounds (**5-9**) | 19-20 |
|  |  |
|  |  |
|  |  |
|  |  |
|  |  |
|  |  |
|  |  |
|  |  |

**The synthesized compounds (5-15**):

|  | |
| --- | --- |
| **Compounds** | **R Group** |
| **5** | -CH_3_ |
| **6** | -C_2_H_5_ |
| **7** | -C_3_H_7_ |
| **8** | -C_4_H_9_ |
| **9** | -C_5_H_11_ |
| **10** | -C_6_H_13_ |
| **11** | -CH(CH_3_)_2_ |
| **12** | -CH_2_-CH(CH_3_)_2_ |
| **13** | -CH_2_-CH_2_-CH(CH_3_)_2_ |
| **14** |  |
| **15** |  |

**Figure S1.** ^1^H NMR spectrum of compound **5**

**Figure S2.** ^13^C NMR spectrum of compound **5**

**Figure S3.** Mass spectrum of compound **5**

**Figure S4.** ^1^H NMR spectrum of compound **6**

**Figure S5.** ^13^C NMR spectrum of compound **6**

**Figure S6.** Mass spectrum of compound **6**

**Figure S7.** ^1^H NMR spectrum of compound **7**

**Figure S8.** ^13^C NMR spectrum of compound **7**

**Figure S9.** Mass spectrum of compound 7

**Figure S10.** ^1^H NMR spectrum of compound **8**

**Figure S11.** ^13^C NMR spectrum of compound **8**

**Figure S12.** Mass spectrum of compound **8**

**Figure S13.** ^1^H NMR spectrum of compound **9**

**Figure S14.** ^13^C NMR spectrum of compound **9**

**Figure S15.** Mass spectrum of compound **9**

**Figure S16.** ^1^H NMR spectrum of compound **10**

**Figure S17.** ^13^C NMR spectrum of compound **10**

**Figure S18.** Mass spectrum of compound **10**

**Figure S19.** ^1^H NMR spectrum of compound **11**

**Figure S20.** ^13^C NMR spectrum of compound **11**

**Figure S21.** Mass spectrum of compound **11**

**Figure S22.** ^1^H NMR spectrum of compound **12**

**Figure S23.** ^13^C NMR spectrum of compound **12**

**Figure S24.** Mass spectrum of compound **12**

**Figure S25.** ^1^H NMR spectrum of compound **13**

**Figure S26.** ^13^C NMR spectrum of compound **13**

**Figure S27.** Mass spectrum of compound **13**

**Figure S28.** ^1^H NMR spectrum of compound **14**

**Figure S29.** ^13^C NMR spectrum of compound **14**

**Figure S30.** Mass spectrum of compound **14**

**Figure S31.** ^1^H NMR spectrum of compound **15**

**Figure S32.** ^13^C NMR spectrum of compound **15**

**Figure S33.** Mass spectrum of compound **15**

**Figure S34.** Computational NMR calculations and DP4+ analysis of compound **5**

**Figure S35.** Computational NMR calculations and DP4+ analysis of compound **9**
